# Mycobacterial Metabolic Syndrome: LprG and Rv1410 Regulate Triacylglyceride Levels, Growth Rate and Virulence in *Mycobacterium tuberculosis*

**DOI:** 10.1371/journal.ppat.1005351

**Published:** 2016-01-11

**Authors:** Amanda J. Martinot, Mary Farrow, Lu Bai, Emilie Layre, Tan-Yun Cheng, Jennifer H. Tsai, Jahangir Iqbal, John W. Annand, Zuri A. Sullivan, M. Mahmood Hussain, James Sacchettini, D. Branch Moody, Jessica C. Seeliger, Eric J. Rubin

**Affiliations:** 1 Division of Immunology and Infectious Diseases, Harvard School of Public Health, Boston, Massachusetts, United States of America; 2 Department of Chemistry, Stony Brook University, Stony Brook, New York, United States of America; 3 Department of Medicine, Division of Immunology and Rheumatology, Brigham and Women’s Hospital, Harvard Medical School, Boston, Massachusetts, United States of America; 4 Department of Chemistry, Texas A&M University, College Station, Texas, United States of America; 5 Department of Cell Biology, SUNY Downstate Medical Center, Brooklyn, New York, United States of America; 6 Department of Pharmacological Sciences, Stony Brook University, Stony Brook, New York, United States of America; National Institutes of Health, UNITED STATES

## Abstract

*Mycobacterium tuberculosis* (*Mtb)* mutants lacking *rv1411c*, which encodes the lipoprotein LprG, and *rv1410c*, which encodes a putative efflux pump, are dramatically attenuated for growth in mice. Here we show that loss of LprG-Rv1410 in *Mtb* leads to intracellular triacylglyceride (TAG) accumulation, and overexpression of the locus increases the levels of TAG in the culture medium, demonstrating a role of this locus in TAG transport. LprG binds TAG within a large hydrophobic cleft and is sufficient to transfer TAG from donor to acceptor membranes. Further, LprG-Rv1410 is critical for broadly regulating bacterial growth and metabolism *in vitro* during carbon restriction and *in vivo* during infection of mice. The growth defect in mice is due to disrupted bacterial metabolism and occurs independently of key immune regulators. The *in vivo* essentiality of this locus suggests that this export system and other regulators of metabolism should be considered as targets for novel therapeutics.

## Author Summary

Of the estimated 2 billion people worldwide currently infected with *Mycobacterium tuberculosis (Mtb)*, surprisingly few go on to develop active tuberculosis (TB) disease. The vast majority, 95 percent, of infected individuals develop latent TB, remaining infected but without disease. Despite its importance in global health, the question of what determines whether an infected individual will develop active or latent TB remains largely unanswered. Changes in how *Mtb* grows in response to stressors presented by the host environment likely play an important role in this process. In particular, the manifold ways in which *Mtb* synthesizes, degrades, and transports lipids dictates its growth in an infected host. Here, we show that lipid transport is an important function of two TB genes known to be required for *Mtb*’s ability to cause disease in the mouse model of infection. Using a variety of genetic and biochemical techniques, we found that the products of these genes prevent the cytosolic accumulation of a lipid associated with non-growing *Mtb* under the metabolic conditions it encounters during infection. Our results indicate an important role for the metabolism of *Mtb* in its ability to orchestrate a productive infection and cause disease.

## Introduction

Tuberculosis continues to be a major global health threat. *Mycobacterium tuberculosis* (*Mtb*) is estimated to infect 2 billion people worldwide, or one-third of the world’s population [1]. Despite its clinical importance, key aspects of tuberculosis (TB) pathogenesis are still not understood, including predictors of whether exposure will lead to active versus latent disease. Only 5% of exposed individuals will go on to develop active disease, whereas the remaining 95% will develop latent disease but remain susceptible to reactivation [2]. Therefore, *Mtb* is able to survive during periods of reduced growth and has the capacity to regrow rapidly.

How does this happen? *Mtb* orchestrates growth arrest in response to stresses encountered in the host, and lipid metabolism, a central part of *Mtb*’s life inside the host, likely plays an integral role [3,4]. While the synthesis and degradation of lipids has been extensively studied, their transport is far less well understood. We have previously identified two genes, *rv1411c* and *rv1410c*, that form an operon and are conditionally essential for survival in the mouse [5,6]. *Rv1411c* encodes the mycobacterial lipoprotein LprG, which can bind triacylated phospholipids such as phosphatidylinositol mannoside (PIM) and lipoarabinomannan (LAM) [7] and is necessary for normal surface display of LAM [8,9]. However, unlike other known mycobacterial lipoproteins, LprG is in an operon with a putative integral membrane transporter, Rv1410, a member of the major facilitator superfamily (MFS) [10,11]. Both genes are required in *M*. *smegmatis* for normal cell wall composition and for efflux of toxins such as ethidium bromide [12]. LprG has structural homology to the mycobacterial lipoprotein LppX, which, along with the transporter MmpL7, is required for the outer membrane localization of phthiocerol dimycocerosate (PDIM), a virulence-associated lipid [13,14]. By analogy, LprG and Rv1410 might function together to position mycobacterial lipids in the cell wall.

LprG binds to PIM and LAM, two TLR2 agonists, and is predicted to transport these two lipids to the cell surface of *Mtb* [7]. Deletion of LprG limits TLR2 activation and blocks certain aspects of phagolysosomal fusion [7,8] but it is unclear whether these effects account for the strong *in vivo* growth attenuation of LprG-deficient bacteria. Loss of TLR2 does not alter growth of *Mtb in vivo* [15] and loss of key components of the TLR-induced signaling cascade, such as MyD88, actually worsen infection due to effects of IFN-γ-mediated activation of macrophages [16,17]. Thus, mislocalization of two TLR2 agonists, PIM and LAM, would not be expected to cause the significant *in vivo* attenuation observed upon loss of LprG function. Given that LprG binds several classes of *Mtb* lipids, at least *in vitro*, we instead posited that the distribution of other lipids within *Mtb* is affected by the loss of LprG and Rv1410.

To investigate this possibility, we used unbiased lipidomic analysis to examine the abundance and distribution of lipids in *Mtb*. We found that disruption of LprG-Rv1410 function leads to an increased level of intracellular TAG. Furthermore, LprG co-crystallizes with TAG and transports TAG between lipid membranes *in vitro*, implying that these proteins are required to transport TAG out of the cytoplasm. This conclusion is supported by data showing that overexpression of LprG-Rv1410 increases release of TAG into the culture medium of broth-grown *Mtb*. Finally, loss of LprG-Rv1410 function is associated with *Mtb* growth attenuation both *in vitro* during carbon restriction and *in vivo*. Thus, we identify LprG as a physiologically important TAG transporter. This previously unknown mechanism likely explains the surprising presence of recently discovered TAG in the outer membrane of *Mycobacterium smegmatis* [18]. Further, because TAG plays an essential role in *Mtb* metabolism [19,20], we propose that an altered metabolic state is likely ultimately responsible for the significantly reduced survival and virulence of LprG-Rv1410 mutants in the host.

## Results

### Loss of LprG-Rv1410 leads to triacylglyceride accumulation in *Mtb*

**Fig 1:**
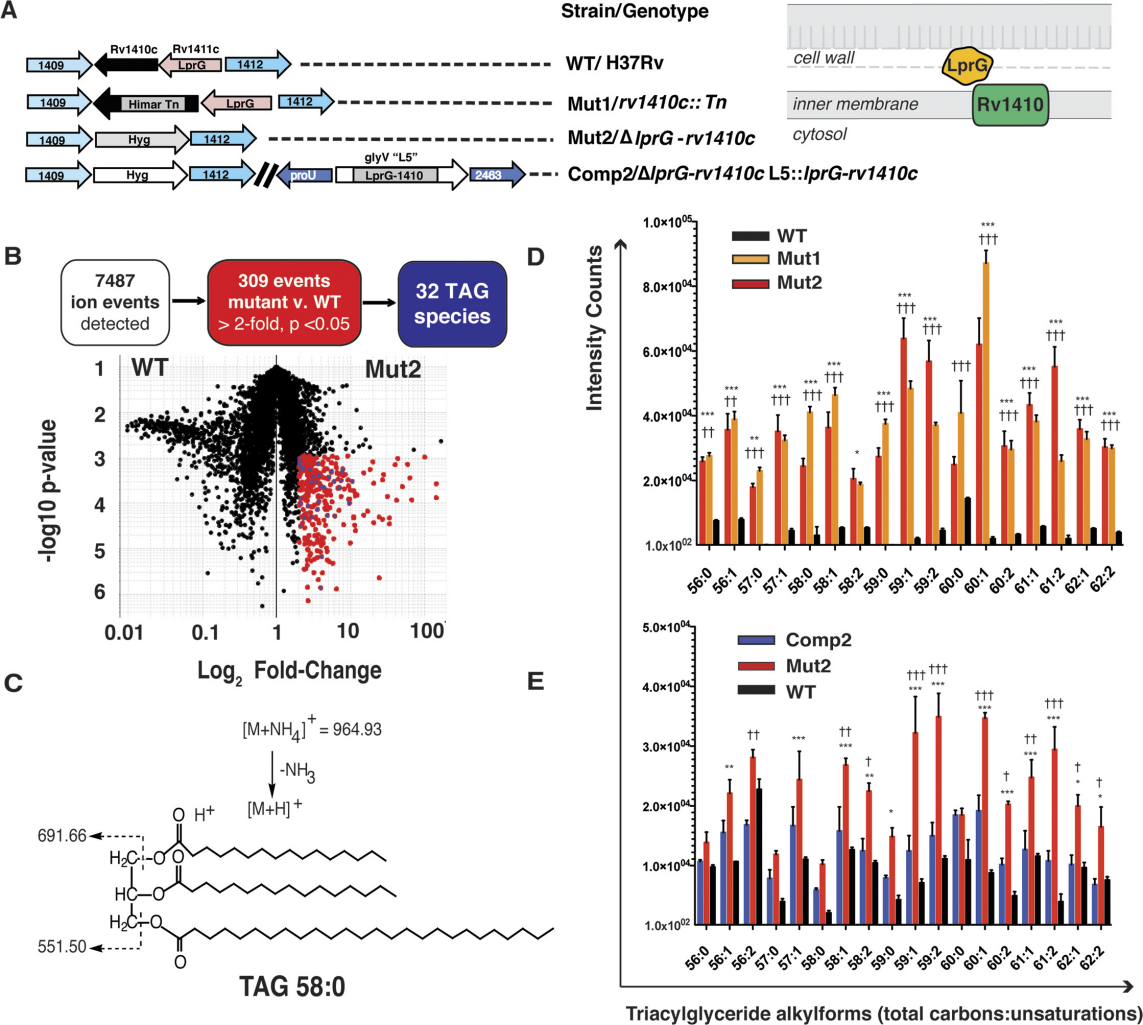
Lipidomics show that loss of LprG-Rv1410 results in triacylglyceride (TAG) accumulation in *Mtb*. (A, left) Strains used in this study. (A, right) Schematic showing predicted localization of proteins encoded by genes manipulated in this study. (B) Positive mode comparative lipidomics analysis from stationary phase *M*. *tuberculosis* Mutant 2 (Mut2) and parental wild-type (WT) strains yielded 7487 total events (black and red) with 309 (red) that meet change criteria of a two-fold increase in Mut2 compared to WT at a corrected p <0.05 (student’s paired t-test, Gene Pattern, Broad Institute). Each dot represents the mean intensity over three biological replicates for a given molecular event in Mut2 compared to WT. For thirty-two of these the *m/z* values match the expected masses of TAG alkylforms (blue) detected as ammonium and hydrogen adducts. (C) Collision-induced dissociation mass spectrometry of a representative TAG (TAG 58:0, *m/z* 564.93). (D-E) Mean intensity values for molecular events mapping to TAG isoforms (ammonium adducts) with the indicated total number of carbons:unsaturations found in all alkyl chains in (D) WT, Mut1 and Mut2 and (E) WT, Mut2, and Complement (Comp2) strains. Error bars show mean +/- SD. * p<0.05, ** p<0.01, ***p<0.001, in (D) Mut1 (†) or Mut2 (*) vs. WT and (E) WT (*) or Comp2 (†) vs. Mut2; 2-way ANOVA with Bonferroni post-test correction.

Our previous studies suggested that LprG and Rv1410 act together in processes that affect cell wall integrity [12]. Thus, we hypothesized that mutations that disrupted one or both genes would have similar phenotypes. We used two different strains: an *Mtb* H37Rv strain with a transposon insertion in *rv1410c* (*rv1410c*::Tn; Mut1) [5] and a strain from which we deleted both genes by allelic exchange (Δ*lprG-rv1410c*, Mut2) (Fig 1A and S1 Fig and S1 Methods). To analyze lipids produced by each strain, we performed comparative lipidomic analyses [21]. Briefly, we extracted cell-associated lipids from *Mtb* during stationary growth phase and analyzed the total lipid content by normal phase high performance liquid chromatography-mass spectrometry (HPLC-MS). We performed initial analyses in both positive and negative ion modes, but, because we found fewer reproducible lipid alterations in negative mode, we focused further experiments on positive mode analysis. We conducted all experiments in biological triplicate and assigned ions appearing in two or more analyses with nearly identical mass-to-charge ratio (*m/z*) and retention time as ‘molecular events.’ By generating mass spectrometry intensity ratios for many thousands of paired events from wild-type and genetically altered mycobacteria, this method provides organism-wide descriptions of lipid change and is capable of identifying individual changed lipids.

For analysis, we aligned all events with equivalent mass and retention time across datasets to enable detection of changed events, which are defined as having an intensity value altered by 2-fold with a corrected p value < 0.05. Among 7487 total events detected, 309 (4.1%) met these criteria. Previous analyses estimate the rate of false positive changed lipids as being less than one percent [21]. The percentage of changed events detected here (4.1%) is above method-specific variance and similar to the percent change detected between the *Mtb* strains H37Rv and W Beijing [21]. The absolute number of changed events exceeded our ability to identify them, so we used previously validated criteria to group and prioritize events for molecular identification [22,23]. In ranking events of known mass but unknown chemical identity, we prioritized those with highest absolute fold-change and lowest p values. Further, in normal phase chromatography, lipid classes are separated in time, but alkylforms within one class show nearly identical retention time with mass intervals characteristic of unsaturations (H2) or alkyl (CH2) chain length variants. In this way, the large list of changed ions can be grouped into a smaller number of lipid classes, even in the absence of knowing the chemical name of each lipid. Within each class, the intensity values of each member can be individually assessed to see whether they change with the same direction and magnitude after mutation. Therefore, as a third criterion for highlighting events of biological interest, we sought lipid classes that showed parallel changes among all events corresponding to the alkylforms in a given lipid class.

Combining these three criteria, we observed a striking pattern for 32 events that all belonged to the same lipid class (Fig 1B, blue). These 32 events increased in intensity with similar magnitude in the knockout strain and matched masses of TAG alkylforms in the MycoMass database. Ion chromatograms showed that the events had the expected retention time of TAG (3–4 min) as annotated in the MycoMap database [21]. The structures of TAG alkylforms with a combined lipid length and saturation in the three chains of 48:0 (*m/z* 824.77), 58:0 (*m/z* 964.94), and 59:1 (*m/z* 976.93) corresponded to the expected total lipid lengths of mycobacterial TAG. In order to formally rule the identity of an event as TAG, we performed collision induced mass spectrometry (CID-MS) and detected the fragments of a protonated adduct of C58 (Fig 1C) and other ions in the series (S2 Fig) as TAG having the expected mass intervals of C16 and C26 fatty acyl units as well as the ions corresponding to the expected diacylglyceride units (*m/z* 691.66, *m/z* 551.50).

### Loss of Rv1410 function is sufficient for triacylglyceride accumulation

Because Rv1410 is a predicted integral membrane protein and LprG is a lipoprotein that is shed into culture medium [24], we hypothesized that Rv1410 functions upstream of LprG in regulating lipid transport across the plasma membrane. To validate this, and to control for potential differences unlinked to the disrupted locus, we performed comparative lipidomics analyses on Mut1 and Mut2 strains to assess the overlap in lipid perturbation phenotypes and focused our analysis on lipids that increased in both mutant strains. By this more stringent analysis, increases in TAG remained the major lipid alterations in both mutant strains. Individual TAG species with differing length (C56-62) and unsaturation (0–2) were detected at statistically significantly elevated levels in both mutant versus wild-type strains across repeat experiments as compared to other detected lipids (Fig 1D and S3 Fig). The absolute fold-change in signals were large, more than 50-fold for most alkylforms, with increases in signal intensity for TAG in Mut1 and Mut2 compared to WT ranging from 2- to 100-fold depending on the TAG alkylform and strain evaluated. Upon complementation with a single copy of the *lprG-rv1410c* operon (Δ*lprG-rv1410c L5*::*lprG-rv1410c*, Comp2), we detected reduced amounts of TAG species compared to Mut2 for every alkylform (Fig 1E).

### LprG binds to triacylglyceride

**Fig 2:**
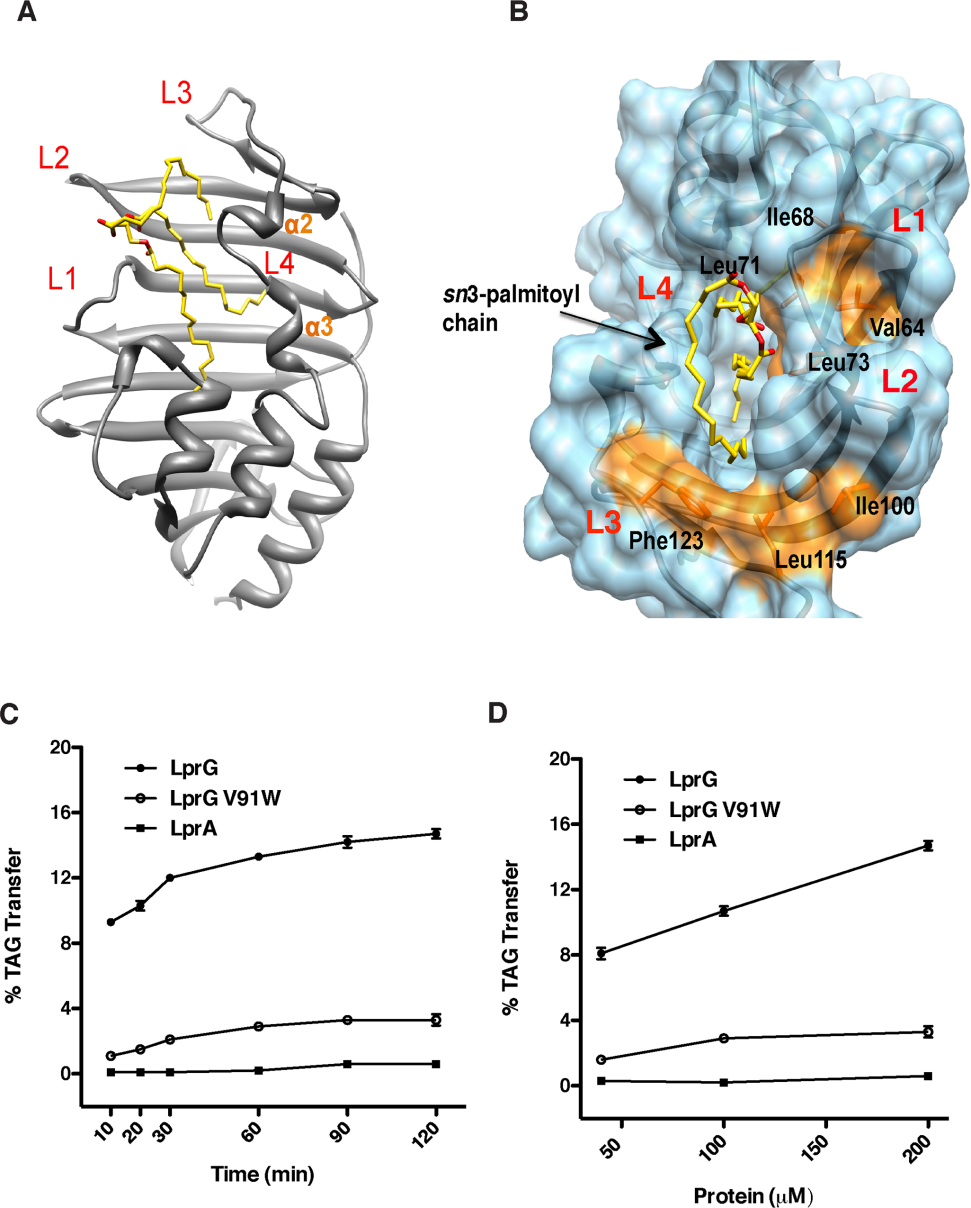
LprG binds and transports TAG *in vitro*. (A) Structure of TAG (48:0) co-crystallized with LprG showing loops L1-L4 that surround the entrance to the binding cavity. (B) Two hydrophobic grooves (orange) on the surface of LprG and close to the binding site for the *sn*3-palmitoyl chain may accommodate TAG with longer acyl chains. (C) Addition of LprG (filled circles) to vesicles with partially self-quenched dye-labeled TAG results in a (C) time-dependent and (D) protein dose-dependent fluorescence increase. Controls include LprG with a bulky mutation V91W in the binding pocket and the homologue LprA. LprG-dependent transfer activity is apparent at the first time point (10 min.) due to lag time between reaction initiation and measurement. Data are the average of three technical replicates and are representative of three independent experiments. Error bars (within data symbols) are +/- SD.

Previous studies have shown that the outer layers of the mycobacterial cell wall contain TAG [25,26]. Further, as shown by reverse micelle extraction of mycobacterial surface lipids, TAG represents a significant proportion of non-covalently associated lipids in the outer membrane of mycobacteria [18]. Based on our lipidomics analysis, we hypothesized that LprG and Rv1410 represent a formerly unknown mechanism to transport TAG from the cytoplasm to the outer membrane. Previous work on the structure of LprG revealed a large central cavity that can accommodate triacylated lipid species, including triacylated phosphatidylinositol (Ac_1_PIM_2_) [7]. Based on a model in which Rv1410 transports TAG across the cytosolic membrane and then transfers TAG to LprG in the cell wall, we hypothesized that LprG should bind similarly to TAG. As predicted, co-crystallization with TAG (tripalmitoylglyceride, TAG 48:0) revealed that the complex (Fig 2A) contains TAG, which is seated similarly to Ac_1_PIM_2_ in the LprG binding site (PDB, 4ZRA). Briefly, the alpha-beta fold is maintained and consists of 10 anti-parallel ß-strands apposed by 6 α-helices that define a central cavity whose entrance is lined by multiple loops (Fig 2A). The TAG glyceryl group is located at this entrance between loops L1 (aa 65–72) and L2 (aa 95–98). Compared to the position of Ac_1_PIM_2_ bound to LprG, the orientation of TAG is conserved: the hydrophilic glyceryl headgroup interacts with Ser72 at the cavity entrance. The acyl chains form interactions with the side chains of hydrophobic residues within the cavity. However, while the three acyl chains of Ac_1_PIM_2_ are all bound within the cavity, only two of the TAG palmitoyl chains (*sn*1 and *sn*2) are similarly buried; the third acyl chain (*sn*3) is exposed to the solvent (Fig 2B). The first ten carbons of the *sn*3 palmitoyl chain form van der Waals contacts with the hydrophobic sidechains at the cavity entrance, including Leu95, Leu115, Phe123 and Ile129. Furthermore, the *sn*3 chain is close to two hydrophobic grooves formed by Phe123-Leu115-Ile100 and Leu73-Leu71-Ile68-Val64 (Fig 2B). This proximity suggests that these grooves could facilitate the binding of TAG with longer acyl chains.

The lid of the cavity, composed of a helix-loop motif (α2-loop1- α3-loop2), is flexible and was found in two conformations that define distinct cavity volumes. The volume of the open form with TAG bound is 1425 Å^3^ and the closed form is 895 Å^3^ due to a major inward movement of the cavity lid (aa 129–134). In the closed form, the hydroxyl group on the side chain of Tyr130 is shifted 4.5 Å toward the center of the cavity, as measured from the hydroxyl oxygen atom on the side chain. Comparing the helix-loop motif in the closed and open forms, the side chain of Tyr130 blocks the binding site for the *sn*2-palmitoyl chain of TAG. Therefore, different conformations of this helix-loop motif may help accommodate the binding of diverse triacylglycerides.

### LprG transports triacylglyceride between lipid bilayer membranes

Given the predicted localization of the two proteins, we hypothesized that Rv1410 transports TAG across the inner membrane and passes it to LprG, which then transfers the lipid to the outer membrane. Thus, LprG should not only bind to TAG but facilitate movement of TAG between lipid membranes, analogous to the suggested role of LppX in PDIM localization to the outer membrane [13]. To test the lipid transfer ability of LprG we used purified proteins in a vesicle-based assay to measure TAG movement between lipid membranes (S4 Fig) [27]. A protein-dependent increase in fluorescence indicates the net movement of fluorescently labeled TAG (dioleoyl-*sn*3-nitrobenzoxadiazole-C_6_ glyceride; NBD-TAG) from donor vesicles containing partially quenched NBD-TAG to acceptor vesicles without TAG and thereby demonstrates whether a protein can both extract TAG from and release it into lipid membranes. We found that LprG was able to transfer lipids in a protein dose-dependent manner (Fig 2C). Also, LprG-V91W, a mutant containing a bulky mutation located within the hydrophobic cavity, transferred TAG at a decreased rate and yield (Fig 2C and 2D) [7]. The marked reduction in activity is consistent with the reported lower binding affinity of LprG-V91W for triacyl lipids. In contrast, the LprG homologue LprA had no detectable activity (Fig 2C and 2D), consistent with our prediction that it cannot bind TAG due to a smaller binding pocket that accommodates only diacylated lipids [7]. Thus, LprG can transfer TAG between lipid membranes, as predicted from its hypothesized role as a lipid transport protein.

### Overexpression of LprG-Rv1410 increases the presence of triacylglycerides in culture medium of *Mtb*

**Fig 3:**
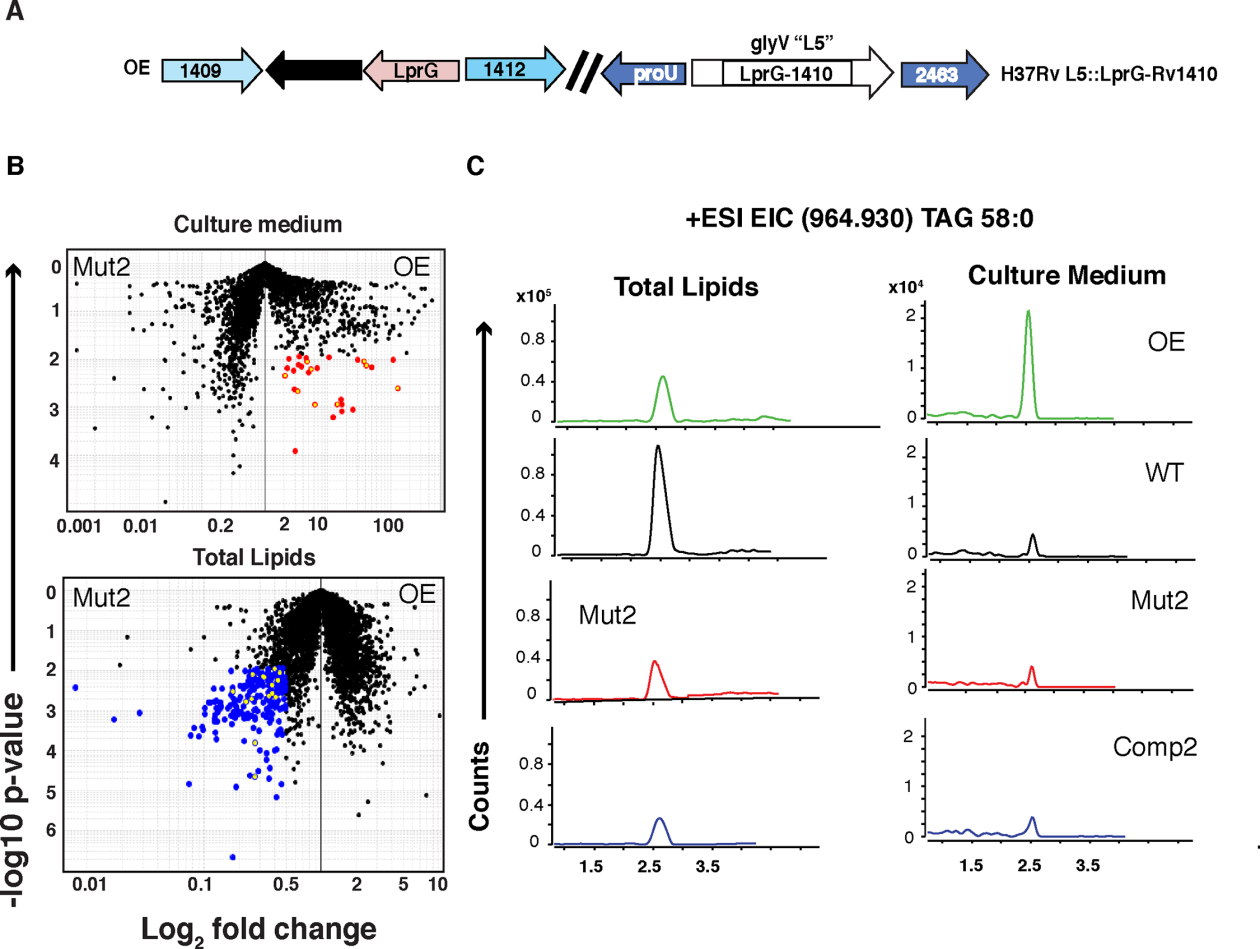
Overexpression of *lprG-rv1410c* increases TAG released into culture medium of broth-cultured cells. (A) Schematic of the overexpression (OE) genotype. (B) Comparative lipidomes of culture medium (upper) and total cells (lower) derived from the OE vs. Mut2 strains from log-phase broth-grown cells (OD 0.7 +/- 0.2). Upper panel, OE vs. Mut2 culture medium (12.5 μg lipid); 2-fold increase, corrected p< 0.05 (red), TAG (yellow overlay). Lower panel, OE vs. Mut2 total cells (12.5 μg lipid); 2-fold decrease, corrected p< 0.05 (blue), TAG (yellow overlay). Student’s paired t-test (Gene Pattern, Broad Institute of Harvard, MIT, MGH). (C) Extracted ion chromatogram (EIC) for *m/z* 964.963 (TAG 58:0) in culture medium versus total cell lipid from OE, WT, Mut2, and Comp2 strains within the same MS experiment.

Recent data suggest that TAG make up a significant proportion of non-covalently associated lipids in the outer leaflet of the outer membrane of mycobacteria [18]. If TAG is transported from the cytoplasm into the cell wall we predicted that it might be shed into culture medium of broth-grown cultures and be detectable by MS. To test this, we compared the quantities of TAG in culture medium derived from both mutant and wild- type strains. Lipidomics analysis of culture medium collected from the WT, Mut2, and Comp2 cells in logarithmic growth phase revealed low signals for lipids shed into the culture medium with few relative differences between the strains in both positive and negative mode analysis. In retrospect, this was not surprising given that we were able to detect differences in total cell lipids between strains from stationary phase, but not log-phase, cultures grown in broth medium. We therefore asked instead whether overexpression of LprG-Rv1410 would lead to increased quantities of TAG in culture medium (Fig 3A and S5 Fig). Indeed, we found a greater signal intensity corresponding to TAG in the culture medium from the overexpression strain (OE) as compared to the LprG-Rv1410 mutant (Mut2) (Fig 3B, upper panel). Importantly, OE had increased TAG in culture medium despite having equivalent to less total cell TAG as compared to Mut2 (Fig 3B, lower panel). The increased shedding of TAG into culture medium was confirmed using extracted ion chromatograms that monitor TAG (58:0) ion counts across all genetically altered and WT mycobacteria (Fig 3C). Furthermore, a trend toward higher normalized TAG signals in the culture medium of OE was seen for each of the individual TAG alkylforms and the difference reached statistical significance for many of the alkylforms. This conclusion was further supported by low detection of the highly abundant inner membrane associated phospholipids, phosphatidylethanolamine (PE) and cardiolipin (CL), indicating that inner membrane and cytosolic lipid did not substantially contaminate the filtered cultured medium. Increased TAG in culture medium of OE is unlikely to be a non-specific effect of overexpression since amounts of control lipids were not increased in culture medium of OE. The identity of TAG and control lipids in culture medium was confirmed by MS/MS fragmentation analysis. Similar results were consistent across two additional experiments that measured TAG accumulation in culture medium of OE across a range of growth conditions (S6 Fig).

### Loss of LprG-Rv1410 function leads to growth attenuation independent of host immune responses

Complementation of the LprG-Rv1410 operon restores virulence of LprG-Rv1410 mutants in mice (S7 Fig) demonstrating that the virulence defect is attributable to loss of function at this specific locus. Previous work suggests that 1) LprG promotes the activation of the innate immune receptor TLR2 through export of lipoglycans such as PIM and LAM [7,28] and 2) plays a role in LAM display [8,9]. Both hypotheses attribute the attenuation of the LprG-Rv1410 mutant in mice to an altered interaction with host immune cells, namely phagocytes, leading to enhanced clearance [8,9]. We reasoned that if either immune mechanism accounted for the strong attenuation seen during infection [29], then loss of innate and acquired immune functions that occur downstream of both of these processes should rescue growth of LprG-Rv1410 mutants *in vivo*.

**Fig 4:**
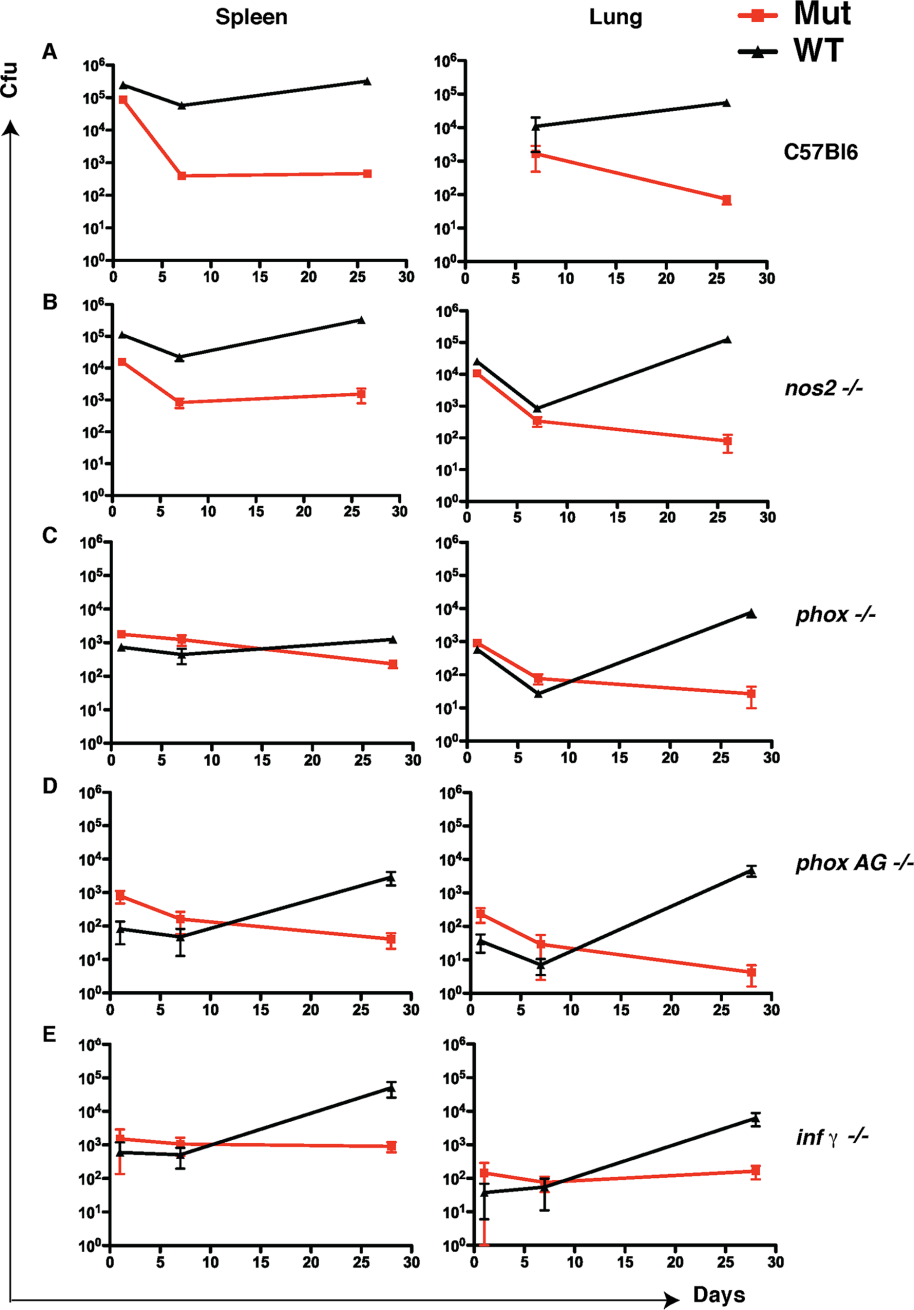
Rv1410 function is necessary for growth of *Mtb* in mice with immune deficiencies, indicating that loss of virulence is intrinsic to the bacterium. Colony forming units (cfu) of WT (H37Rv, black) and *rv1410*::Tn (Mut1, red) from spleen and lungs of mice infected with a 3:1 (Mut1:WT) mixture. (A) C57/Bl6 (B) *nos2-/-* (C) *phox-/-* (D) *phox-/-* fed aminoguanidine *ad libitum*, and (E) *inf-γ -/-* mice. Cfu were measured in lung and spleen at 1, 7, and 30 days post infection. Error bars show mean +/- SD of three to five animals per time point.

To examine the role of the innate immune response, we infected *nos2-/-* and *phox-/-* mice that are compromised for oxidative killing mechanisms and neutrophil function, respectively. Oxidative killing by nitric oxide generated by inducible nitric oxide synthase (iNOS; *nos2*) in macrophages and neutrophils is a well-documented mechanism for *Mtb* control in mice [30–32]. Similarly, NADPH oxidase (Phox; *phox*) is required for reactive oxygen species (ROS) killing of mycobacteria by neutrophils and macrophages downstream of TLR signaling in response to infection [33,34]. C57/Bl6 wild-type and mutant mice were infected with a 3:1 mixture of Mut1 and the matching H37Rv WT background control. Mut1 was attenuated in the lung and spleen in all mouse strains, as assessed by colony forming units (cfu) (Fig 4A and 4B and 4C). Furthermore, loss of generalized oxidative killing also failed to rescue growth of Mut1 in *phox -/-* mice administered the iNOS inhibitor aminoguanidine (Fig 4D). Thus, the attenuation of a mutant lacking Rv1410 activity is independent of these innate immune control mechanisms.

The onset of the adaptive immune response initiates secondary control mechanisms against *Mtb* growth. Specifically, the pro-inflammatory cytokine IFN-γ is critical for *Mtb* infection control in both mice and humans [35–37]. Importantly, *Mtb* can downregulate host immune responses by dampening classical macrophage activation and IFN-γ mediated effector functions via TLR2 signaling [38]. If the absence of lipid bound-LprG in LprG-Rv1410 mutants results in attenuation due to a shift towards classical macrophage activation, then we predicted that the growth of these mutants should be rescued in *ifn-γ -/-* mice. We infected *ifn-γ* -/- mice with mixed Mut1 and WT *Mtb* and found that the mutant strain was again attenuated relative to WT even though three times the infective dose of Mut1 vs. WT was delivered (Fig 4E).

**Fig 5:**
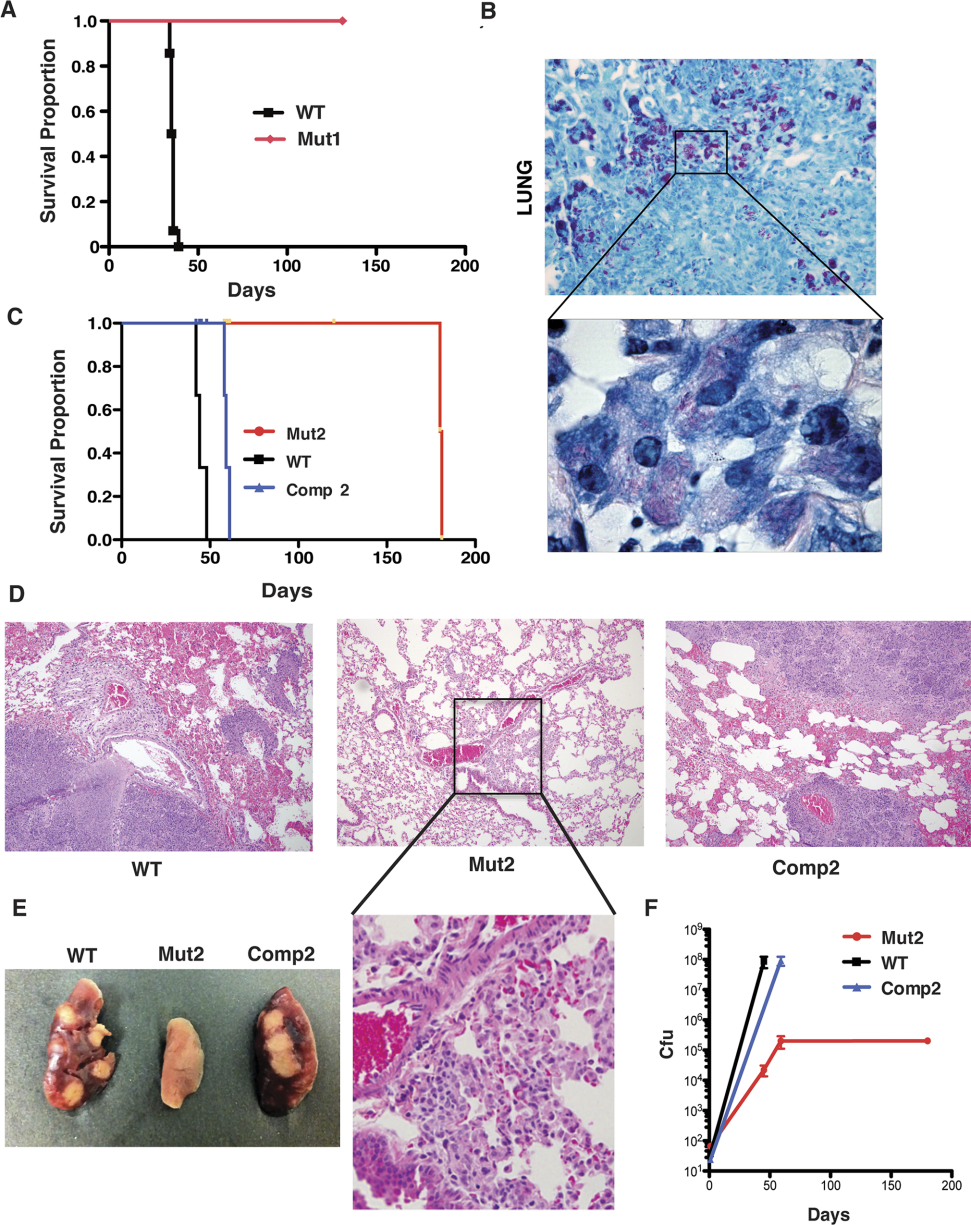
Attenuation of the *Mtb* LprG-Rv1410 mutant in mice persists in the absence of adaptive immunity and is associated with slow growth. (A) Survival of 28 *rag -/-* mice infected with either Mut1 (red, n = 14) or WT (black, n = 14) and followed for 5 months. (B) Representative lung section from a Mut1-infected mouse at end of study (Day 150) with numerous acid-fast mycobacteria, 400x magnification; (inset) multibacillary growth of Mut1 in lung macrophages, 1000x oil magnification. Ziehl-Neelsen acid-fast with methylene blue counterstain. (C) Survival of 21 SCID mice infected via aerosol with 50–100 cfu of WT (black, n = 5), Mut2 (red, n = 11), or Comp2 (blue, n = 5) followed for six months. WT- and Comp2-complement infected mice died by 60 days post-infection. Mut2-infected mice died at 6 months post-infection. (D) Three Mut2- infected mice were sacrificed at the time of death of WT- and Comp2- infected mice. Hematoxylin and eosin (H&E) stain of lung from mice infected with WT (left), Mut2 (middle) and Comp2 (right) at time of death (WT and Comp2) or 60 days (Mut2) post-infection, 200x magnification; (inset) small foci of macrophage infiltrate in Mut2-infected lung, 600x magnification. (E) Formalin- fixed lungs from infected SCID mice. WT (left), Mut2 (middle), Comp2 (left) showing tan nodules on the surface of both WT and Comp2 lungs. (F) Lung cfu from SCID mice infected with WT, Mut2, or Comp2.

IFN-γ is only one of many effectors that might play a role downstream of adaptive immunity. To more comprehensively test the role of the host adaptive immune response in controlling infection with LprG-Rv1410 mutants we used *rag-/-* or severe combined immunodeficiency (SCID) mice, which both lack functional B and T lymphocytes. Rag-deficient mice succumbed to disease at 3 weeks post infection with WT bacteria, whereas Mut1-infected mice were still alive at 5 months post infection when the experiment was terminated (Fig 5A). At the end of the experiment, three Mut1-infected mice were moribund with numerous acid-fast bacilli in their lungs (Fig 5B). This was not specific to the bacterial mutant, the mouse strain or the route of infection, as SCID mice infected with the Mut2 strain by the low-dose aerosol route also survived for an extended time (Fig 5C). Thus, the attenuation of the LprG-Rv1410 mutant is seen in mice with variously altered immune responses (S1 Table), including innate and downstream adaptive mechanisms that are thought to be relevant to PIM and LAM action.

### Growth of *Mtb* lacking LprG-Rv1410 is slow during infection

If loss of LprG-Rv1410 interaction with host immunity does not explain lack of growth of LprG-Rv1410 mutants *in vivo*, why are these bacteria so markedly attenuated? One clue comes from longitudinal histopathologic examination of organs harvested from immunocompromised mice infected with LprG-Rv1410 mutants. We sacrificed a subset of Mut2-infected SCID mice at time points matching the time of death of WT- and Comp2-infected animals. At earlier time points (60 days) there were few bacteria and little pathologic change in tissues of Mut2-infected lungs, by gross and microscopic pathology (Fig 5D and 5E). Mut2-infected lungs showed only early evidence of thickening of the alveolar septa due to an influx of macrophages (Fig 5D, middle and inset). Histiocytic consolidation, suppuration, and necrosis associated with bacterial replication and/or cytotoxic response were not evident in Mut2-infected mice, as seen in WT and Comp2-infected mouse lungs at the time that mice became moribund (Fig 5D, left and right). Cfu from the lungs of SCID mice infected with the LprG-Rv1410 mutant were 2 log-fold lower in mice infected with Mut2 at matched time points compared to both WT- and Comp2-infected mice (Fig 5F). However, after more prolonged infection, bacterial load increased systemically and the histopathology in Mut2-infected moribund mice was similar to that seen at early time-points with WT or Comp2 *Mtb* strains at time of death. Cfu from the spleen of Mut2- infected mice when moribund, although still lower than for WT and Comp2 when moribund, were nearly 1x10^6^. At six months post-infection aggregates of viable and necrotic macrophages containing numerous acid-fast bacilli were present in the lung, spleen, heart, and kidney of Mut2-infected SCID mice, consistent with the perimortem changes described above for WT- and Comp2-infected SCID mice at 50–60 days post-infection (S8 Fig). These results suggest that *Mtb* lacking LprG-Rv1410 induce the same host reaction as WT but take longer to accumulate the bacterial mass necessary to induce pathologic changes. Comparison of the growth trajectory as measured by cfu over time across the three strains suggests Mut2 grew more slowly during infection, again pointing away from host response as the major mechanism by which LprG-Rv1410 affects *Mtb* virulence.

### *Mtb* lacking LprG-Rv1410 function grows slowly under *in vitro* conditions that mimic infection

**Fig 6:**
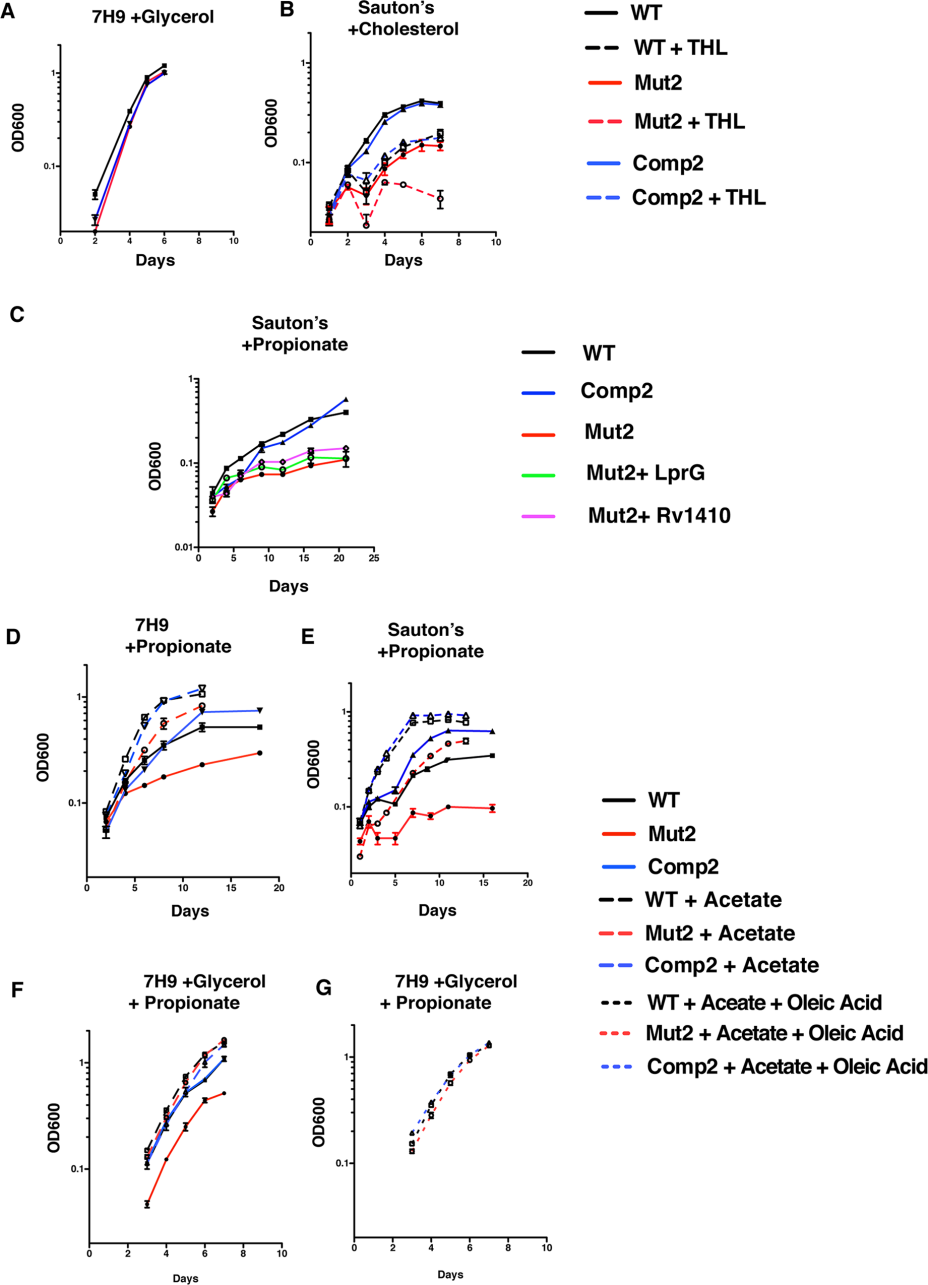
Carbon restriction slows growth of LprG-Rv1410 mutants *in vitro* and phenocopies slow growth *in vivo*. WT, Mut2, and Comp2 were cultured in (A) 7H9 plus ADC (albumin, dextrose, catalase) supplement, 0.2% glycerol, and 0.05% Tween80; (B) Sauton’s minimal medium plus 0.01% cholesterol, 0.05% Tyloxapol and with (dashed) or without (solid) tetrahydrolipostatin (THL; 5 μg/mL); (C) Sauton’s minimal medium, 10mM propionate; (D) 7H9 with ADC, 0.05% Tween80, 10 mM propionate with (large dashed) or without (solid) 5 mM acetate; (E) Sauton’s minimal medium plus 0.05% Tyloxapol, 10mM propionate with (large dashed) or without (solid) 5 mM acetate; (F) 7H9 plus ADC, 0.2% glycerol, 10 mM propionate with (large dashed) or without (solid) 5 mM acetate; (G) 7H9 plus ADC with 10 mM propionate, 5 mM acetate, and 0.05% oleic acid (small dashed). (F,G) represent one experiment. Growth was measured as OD600 at the indicated times. Generation time during exponential growth was estimated from the log-linear slope (S2 Table). Data are the average of three technical replicates and are representative of three independent experiments. Error bars (within data symbols) are +/- SD. Mut2+ LprG (*ΔlprG-rv1410c* L5::*lprG*); Mut2+ Rv1410 (*ΔlprG-rv1410c* L5::*rv1410c*).

Although LprG-Rv1410 mutants grow slowly in the host environment, they exhibit no growth defect *in vitro* in standard culture medium (Fig 6A and S2 Table). Furthermore, we were unable to detect reproducible differences between wild-type and LprG-Rv1410 mutant strains with *in vitro* infections of several murine monocytic cell lines. One potential explanation for this dichotomy is that *Mtb* utilizes lipids as a carbon source during infection, while glycerol is the predominant carbon source provided in artificial medium *in vitro* [39]. Thus, we tested whether the growth of *Mtb* mutants would be slower when grown *in vitro* with lipids as the sole carbon source. Indeed, Mut2 had a reduced growth rate when cholesterol was the primary carbon source (Fig 6B and S2 Table). Growth rate attenuation was even more striking on propionate, a toxic byproduct of cholesterol and fatty acid metabolism [40,41] (Fig 6C and 6D and 6E and S2 Table). The growth defect was not reversible with complementation of either LprG or Rv1410 alone (Fig 6C). Vitamin B12 (VitB12) supplementation also failed to rescue the growth of Mut2, suggesting that the deficiency is unlikely a result of a defect in the methylmalonyl pathway of propionate detoxification (S9 Fig). Therefore, the LprG-Rv1410 mutant displays an *in vitro* growth defect when grown on lipids similar to those seen during infection.

### Intracellular TAG accumulation suppresses *Mtb* growth

We hypothesized that the growth defect of Mut2 when the carbon source is restricted to lipids is the result of intracellular TAG accumulation and that blocking TAG transport via the loss of LprG-Rv1410 function enhances TAG accumulation. If altered levels of intracellular TAG in LprG-Rv1410 mutants can account for their growth defect *in vivo*, we predicted altered growth kinetics of LprG-Rv1410 mutants *in vitro* due to modified TAG levels. Given the large numbers of genes encoding both TAG synthases and lipases in mycobacteria [42,43], genetically altering TAG metabolism proved difficult, so we opted to modulate TAG levels pharmacologically by inhibiting TAG hydrolysis. We predicted that preventing TAG breakdown would exacerbate the growth defect of Mut2. Treatment with tetrahydrolipostatin (THL) decreases TAG hydrolysis by lipases such as LipY, which is thought to mobilize stored TAG as an energy source during the transition out of hypoxia [20]. During growth with cholesterol as the sole carbon source, THL at 5 μg/ml was bacteriostatic during the first 5 days of culture for all strains tested (Fig 6B). The growth rate of the WT and Comp2 subsequently recovered and, interestingly, mirrored the growth rate of Mut2 in the absence of THL. In contrast, the Mut2 treated with THL did not grow throughout the 7-day culture period.

Conversely, TAG hydrolysis by lipases is thought, via β-oxidation, to provide acetyl Co-A for both lipid synthesis via FasI and anapleurosis of the TCA cycle [44,45]. Therefore we predicted that bypassing the need for TAG hydrolysis should at least partially relieve the LprG-Rv1410-dependent growth defect. To test this, we supplemented the medium with acetate, which has been shown to rescue the growth of *Mtb* mutants with defects in the methylcitrate cycle or in cholesterol metabolism when grown in the presence of propionate or cholesterol, respectively [46,47]. As predicted, acetate partially rescued the growth of Mut2 on propionate in both minimal and rich medium (Fig 6D and 6E and S2 Table). Similarly, the addition of glycerol and/or oleic acid rescued growth kinetics of Mut2, which under these conditions were indistinguishable from those of WT and Comp2 strains (Fig 6F and 6G and S2 Table). Furthermore, we determined that the LprG-Rv1410 mutant has decreased susceptibility to THL in medium supplemented with glycerol and oleic acid (S10 Fig and S1 Methods). Therefore, although inducing TAG accumulation mimics or exacerbates the loss of LprG-Rv1410 function, mimicking TAG hydrolysis by supplementing TAG breakdown products such as fatty acids and glycerol has the potential to relieve this stress and restore growth. Growth rescue by acetate, glycerol, and free fatty acids (FFA) is likely mediated by anapleurosis of the TCA cycle, but in the context of TAG accumulation, increased levels of these metabolites may also override inhibitory effects of intracellular TAG by relieving numerous potential negative feedback mechanisms, on processes ranging from fatty acid synthesis to cell cycle regulation and cell division, that are only beginning to be described in mycobacteria [48–52].

## Discussion

Mycobacteria, and *Actinomycetes* in general, are unique not only in their capacity to synthesize and store large quantities of TAG, but also in their ability to catabolize TAG as an energy source during starvation [19,53] [54]. TAG accumulates within lipid droplets in the bacterial cytoplasm [42,55] and is associated with slow growth and antibiotic tolerance (52). TAG in the form of lipid droplets provides energy via β-oxidation of the acyl chains [45,56] and TAG seems to serve a structural role as a major component of the outer leaflet of the outer membrane [18]. Like PDIM, TAG can also serve as a sink to alleviate the accumulation of potentially toxic propionyl-CoA [46,57]. Here we show loss of LprG and Rv1410 disrupts steady-state levels of intracellular TAG in *Mtb*. This could result in multiple cellular defects, consistent with the numerous roles that TAG is proposed to play in mycobacterial physiology and metabolism.

**Fig 7:**
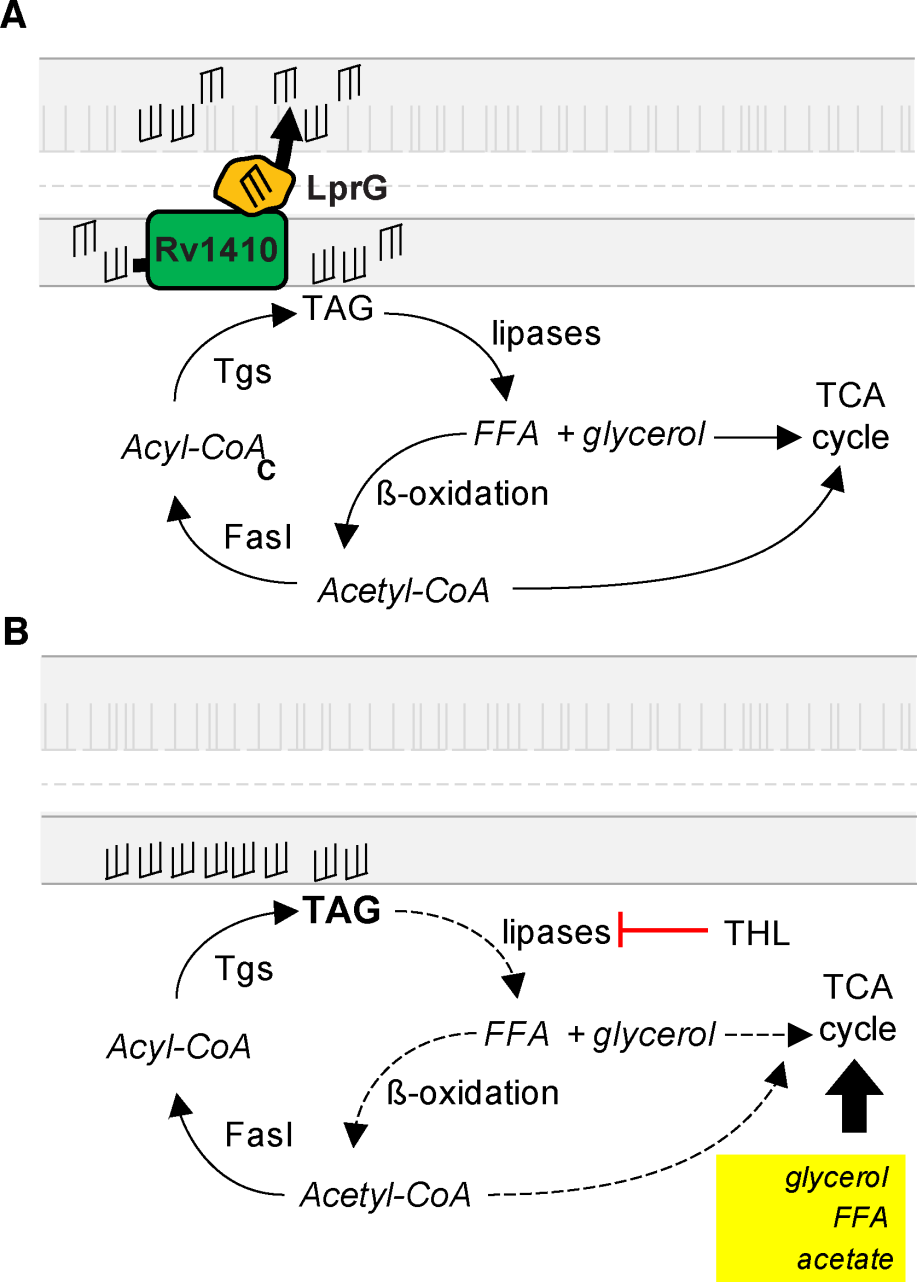
Model for the role of LprG-Rv1410-mediated TAG transport in regulating the growth rate of *Mtb*. (A) In the presence of a functional LprG-Rv1410, FasI makes acyl primers (acyl-CoA) for cell wall lipid synthesis (including TAG via TAG synthases, *Tgs)*. TAG can then be 1) incorporated into the *Mtb* cell wall via the action of LprG-Rv1410 or 2) hydrolyzed by lipases. Acetyl-CoA for anapleurosis of the TCA cycle is generated via β-oxidation of free fatty acids (FFA) liberated from TAG. (B) Loss of LprG-Rv1410 function results in increased intracellular TAG due to lack of transport into the cell wall. The presence of accumulated intracellular TAG may inhibit growth by a currently unknown mechanism. Decreasing lipolysis via lipase inhibitors further increases intracellular TAG levels. Addition of acetate, glycerol, or FFA bypasses the need for lipolysis by providing acetyl-coA that feeds into the TCA cycle and relieves growth arrest.

Similar to a recent report [58], we show that loss of LprG-Rv1410 results in marked slowing of growth *in vitro* when *Mtb* are restricted to lipid carbon sources, such as cholesterol, that are likely utilized during infection. Others have shown this gene defect causes immune-activating defects *in vitro*, but several lines of evidence suggest that the metabolic defects associated with LprG-Rv1410 loss play a larger role in growth attenuation *in vivo*. In the host, lipids represent the major carbon sources available to the pathogen and these conditions favor TAG production [59]. We propose that, under such conditions, wild-type *Mtb* can either transport TAG out of the cytoplasm into the outer layers of the cell wall or store intracellular TAG, the subsequent hydrolysis of which generates energy via anapleurosis of the TCA cycle and enables growth (Fig 7A). Indeed, the growth defect of the LprG-Rv1410 mutant can be titrated by chemical modulation of TAG lipolysis using THL or by providing alternative catabolites, such as acetate and glycerol. The modulation of the growth defect under such conditions is thus likely a result of altered metabolism.

In our model cytosolic TAG accumulation restricts bacterial growth and LprG-Rv1410-mediated transport of TAG into the cell wall functions as an adjunct to lipases to eliminate cytosolic TAG. In mycobacteria stresses such as acidic pH, carbon restriction, acid stress and hypoxia induce shifts in central carbon and lipid metabolism in *Mtb* that lead to intracellular TAG accumulation [4,19,42,44]. Under these conditions there has typically been an association of intracellular TAG with an altered growth state. We present a model by which TAG may chemically or physically participate in growth regulation during the carbon restriction experienced in the host. One possibility is that cytosolic TAG levels in mycobacteria contribute to negative feedback mechanisms known to regulate lipid metabolism in other bacterial species. For example, FFA act as regulatory molecules in *E*.*coli* lipid biosynthesis [60] and recent data suggest that FFA regulate lipid synthesis by regulating fatty acid synthase (FasI) in both mycobacteria and corynebacteria [48,61,62]. FFA have also recently been shown to bind transcription factors that regulate the MmpL family of transporters, some of which transport specific classes of outer membrane lipids and are also associated with lipid biosynthesis [63]. If FFA released by TAG hydrolysis act in a similar fashion, TAG transport out of the cytosol and into the outer membrane could allow for sequestration of these lipids as a means to regulate central carbon and lipid metabolism in mycobacteria.

One alternative explanation for our findings is that loss of LprG-Rv1410 results in increased TAG production. However, increased TAG production occurs normally during the course of wild-type *Mtb* infection, as seen with lipid body formation, and is therefore unlikely to account for the attenuation of LprG-Rv1410 mutants in mice. The model presented here suggests that TAG transport by LprG-Rv1410 is critical under conditions that favor increased TAG production and storage during cellular stress. During growth resuscitation after hypoxic stress, intracellular stores of TAG are hydrolyzed, providing acetyl-CoA to fuel the TCA cycle. A similar dynamic likely occurs during carbon restriction. We propose that titration of growth in response to carbon restriction is partly a function of intracellular TAG levels and that these levels are modulated by TAG export and/or hydrolysis. In the background of LprG-Rv1410 loss, this balance is shifted towards TAG accumulation and therefore bacterial growth arrest is sustained (Fig 7B). It has been proposed that one way in which mycobacteria can co-catabolize multiple carbon sources is by segregating lipid catabolism into different compartments within the cell, including the periplasmic space [64]. One possibility is that TAG transport by LprG-Rv1410 contributes to carbon source segregation in mycobacteria. Our model can account for the attenuation and slow growth rate seen with the LprG-Rv1410 mutant in SCID mice and the overall lack of growth rescue irrespective of host immune status.

In this study we set out to uncover the mechanism responsible for the *in vivo* attenuation reported for both LprG and Rv1410 mutants in mice [5]. To date, much of the data on the role of the *lprG-rv1410c* operon has centered on LprG, an experimentally more accessible molecule. For the phenotypes we have tested in *Mycobacterium tuberculosis* and *Mycobacterium smegmatis* [12], LprG mutants essentially phenocopy Rv1410 mutants, suggesting that these proteins act in concert to perform an essential function during growth within the host. Here we report that LprG binds TAG and is capable of mediating lipid transfer between vesicles. Thus, it appears likely that Rv1410 is responsible for the transport of TAG across the cytosolic membrane, allowing LprG to access TAG, although we cannot measure this directly. This model predicts that mislocalization of TAG would contribute to the increased levels detected in LprG-Rv1410 mutants. Our studies evaluating TAG released into culture medium show that overexpression of LprG-Rv1410 increases the presence of TAG, further supporting our hypothesis that TAG is a quantitatively significant substrate of the LprG-Rv1410 transport system.

Is TAG transport the only role for the LprG-Rv1410 system? Other reports show that various lipids can bind to LprG *in vitro* and that cells that lack this transporter system display reduced levels of surface LAM [8,9]. TAG, PIM and LAM are triacylated lipids that are all proven to bind and occupy the large cavity of LprG, suggesting that the system operates to control the localization of triacylated lipids. [8,9]. However, upon disruption of the LprG-Rv1410 locus, steady-state quantities of TAG change more than 50-fold, which is much greater than the changes observed for the immunogenic phospholipids PIM and LAM. Demonstration of differential localization of TAG within the cell wall will be necessary to confirm our model that LprG-Rv1410 physically transports TAG. Nevertheless, our data suggest that the conditional essentiality of the *lprG-rv1410c* operon *in vivo* is a direct consequence of its non-redundant role in modulating intracellular TAG and, thereby, lipid metabolism and growth regulation.

TAG storage has long been a defining feature of mycobacterial infection. TAG in *Mtb* has served primarily as a marker for stationary, dormant, or latent states, all of which have implications for drug effectiveness, drug resistance, and disease progression. Yet, we have only rudimentary knowledge about how TAG contributes to the processes leading to these states and overall cellular metabolism at the host-pathogen interface, an understanding of which is critical to the development of new preventive and therapeutic approaches to infection control. We propose that TAG transport critically regulates intracellular TAG levels during certain growth states within the host [19,42,55,65]. Certainly, lipid metabolism is central to growth regulation in mycobacteria. Our model suggests that synthesis, transport, and hydrolysis of TAG are balanced in a manner that regulates growth rate, assigning a far more complex role for TAG in *Mtb* cellular homeostasis than has been previously suggested. Clearly, though, regulation is more than just abundance, and future studies will need to evaluate the subcellular distribution of TAG and other lipid mediators in order to determine their collective roles in lipid homeostasis and potential growth regulation.

## Materials and Methods

### Ethics statement

The Harvard Medical School (HMS) IACUC approved the animal care and use protocol 03000. The HMS IACUC adheres to the Public Health Service Policy and Animal Welfare Act.

### Culture of mycobacteria

The virulent H37Rv strain of *Mtb* was used for all experiments unless otherwise noted. Cells were routinely cultured in Middlebrook 7H9 broth and supplemented with 10% (vol/vol) OADC (Middlebrook), 0.2% glycerol, and 0.05% Tween 80. For cells destined for lipidomics analysis, Tween-free media was used. Cells were maintained at 37°C with shaking at 100rpm.

### Lipid extraction, normalization, and high performance liquid chromatography mass spectrometry

Lipids were extracted and analyzed as previously described from *Mtb* during either logarithmic growth phase in 7H9 liquid medium (O.D. 0.8 +/- 0.1) or stationary growth phase from 7H9 agar plates (1.5% w/v Bacto-Agar) [21]. For extraction of lipids from culture medium, approximately 50 mL of culture medium obtained after centrifugation of *Mtb* grown in Tween-free medium was filtered twice with 1 μm average pore-size filters (Millipore). Lipids were extracted by addition of 6 N HCl (0.3% final v/v) followed by addition of 140% v/v ethyl acetate for 30 minutes with gentle rocking. Extracts were centrifuged and the upper organic phase collected, pooled, and evaporated to dryness using a Genevac system (SP Scientific). Lipid masses per sample were measured and dried lipids were resuspended to 1mg/mL in 1:1 chloroform: methanol. 5 μg to 20 μg of total lipid per technical and biological run were injected for HPLC/MS analyses. Sample titration was necessary to determine sample load for optimal detection and quantitation of TAG using normal phase chromatography. Lipids events were detected and defined as previously described using positive mode HPLC/MS [21], and lipidomes were analyzed using XCMS algorithm in R (Scripps) and GenePattern Software (Broad Institute). See also supplemental experimental procedures.

### Triacylglyceride transfer assay

C-terminally 6xHis-tagged recombinant proteins were cloned, overexpressed, and purified largely as described (Suppl Experimental Methods) [28]. TAG transfer assays were performed as previously described [27]. Briefly, donor vesicles containing 450 nmol/mL phosphatidylcholine (PC) and 14 nmol/mL of fluorescently labeled NBD-TAG (dioleoyl-*sn*3-nitrobenzoxadiazole-C_6_ glyceride, custom synthesis by Avanti Polar Lipids) were mixed 1:1 with acceptor vesicles containing 2400 nmol/mL of PC either with or without LprG, LprG-V91W (LprG-VW), or LprA and buffer for a final volume of 100 μL. Plates were incubated at 37°C and fluorescence was monitored as a function of time with a 7620 Microplate Fluorometer (Cambridge Technology, Watertown, MA) using 460-nm excitation and 530-nm emission. The fluorescence representing the total TAG content of donor vesicles was determined by disrupting donor vesicles with isopropanol and measuring the final fluorescence. Percent TAG transfer was determined by subtracting the no-protein control and dividing by total TAG content [27]. See also supplemental experimental methods and S4 Fig.

### Co-crystallization with triacylglyceride

Purified LprG was produced as described previously [7]. Prior to the crystallization, a three-molar excess of powdered tripalmitoyl glyceride (TAG) was directly added to LprG. The mixture was incubated on ice for 1 h. Crystals were grown in 0.1 M sodium acetate buffer pH 4.5 with 25% (w/v) PEG3350. Data were collected to a resolution of 1.8 Å at the Argonne National Lab (beamline 23-ID). Processed with HKL2000, the space group was C2 and cell dimensions were a = 95.7 Å, b = 71.6 Å, c = 61.8 Å, α = 90°, β = 106.6°, γ = 90°. Structural solution was obtained by MOLREP, using the apo-LprG structure (PDB: 3MH8) as an initial model. The structure was built and refined with coot, CCP4 and PHENIX. The final R-work and R-free were 20.4% and 23.4%, respectively.

### Mouse infections

Six- to eight-week-old mice were purchased from The Jackson Laboratory (Bar Harbor, ME). The various mouse strains and their relevant immunological characteristics are shown in S1 Table. Mice were housed under sterile conditions in an ABSL3 facility, and all animal experiments were performed under an animal protocol approved by Harvard University. Mycobacterial strains were grown in 7H9 with OADC (Middlebrook), 0.2% glycerol and Tween 80. Approximately 1x10^6^ cfu were administered for IV infections. All strains used for mouse infections (WT, Mut1, Mut2, Comp2) were confirmed PDIM positive by TLC. For aerosol infections cells in log-phase growth were sonicated and diluted 1:100 in PBS, and approximately 100–300 cfu administered. For competition experiments the mutant *Rv1410*::*Tn* (Mut1), and wild- type, H37Rv (WT), strains were mixed at a ratio of 3:1 (Mut1:WT). WT is unmarked and Mut1 is kanamycin-resistant. For all experiments, spleens and lungs were harvested and plated for cfu on 7H10 agar or Middlebrook 7H9+1.5% Bacto Agar (Difco) containing OADC enrichment (Middlebrook) and 0.2% glycerol with and without kanamycin (25 μg/mL). Three to five mice per group were harvested at the indicated time points. A subset of *phox-/-* received the reagent aminoguanidine (AG) *ad lib*. For survival of *rag-/-* mice, 14 mice per group were infected with WT or Mut1 and followed out to five months. For survival of SCID mice, double *lprG-rv1410c* knockout (Mut2) and complement strains (Comp2) were generated as described (S1 Fig and S1 Methods). Five mice per group were infected by aerosol and followed for 6 months. An additional 10 SCID mice were infected with Mut2 and followed longitudinally with matched sacrifices at time of death of WT infected mice and Comp2-infected mice. At time of death complete necropsy, histopathological evaluation, and cfu enumeration from lung and spleen homogenates were performed. See also supplemental experimental methods.

### Carbon restriction

Mycobacteria were cultured as described above. For carbon restriction, Sauton’s minimal media containing 0.5 g/L asparagine, 1.0 g/L KH_2_PO_4_, 2.5 g/L Na_2_HPO_4_, 50 mg/L ferric ammonium citrate, 0.05g/L MgSO_4_•7H_2_0, 0.05 g/L CaCl_2_, 0.01 mg/L ZnSO_4_ and 0.05% Tyloxapol (vol/vol) was used with addition of either cholesterol (0.01% w/v) or propionate (10 mM) with or without supplementation with acetate (5 mM), glycerol (0.2% v/v), or both, as indicated. Cholesterol-containing media was prepared from 500x stocks made by dissolving cholesterol in 50:50 ethanol/Tyloxapol.

## Supporting Information

S1 Methods Supplementary Methods includes further information on 1) Genetic Manipulation of *Mtb*, 2) Data processing, quantitative data analysis and statistical analysis using R and Gene Pattern, 3) Cloning and expression of lipoprotein expression vectors, and purification of His_6_-tagged lipoproteins, 4) Determination of MIC for tetrahydrolipostatin, and 5) Histopathologic analysis of mouse tissue. (PDF)

S1 Fig (Related to Fig 1 and S1 Methods) Genetic manipulation of *Mtb* to generate LprG-Rv1410 double knockout. The cosmid 2_33 containing the *lprG-rv1410c* genes was used as a template to amplify a linear PCR product (ajm2F, ajm2R) with 50 basepair (bp) homology upstream and down stream of the operon flanking a chloramphenicol/hygromycin (Chlor/Hyg) resistance cassette. Step (1) Recombineering was performed in the *E*.*coli* pkD119 strain containing a lambda-red recombinase plasmid to exchange the Chlor/Hyg cassette with the *lprG-rv1410c* genetic locus on the 2_33 cosmid. Step (2) the recombinant cosmid is used as a template to generate a linear PCR product (ajm3F, ajm3R) with 500 bp chromosomal homology upstream and downstream of the *lprG-rv1410c* operon (replaced by Chlor/Hyg). Step (3) the 3kb linear PCR product is transformed into confirmed PDIM positive *Mtb (H37Rv)* previously transformed with pRecET, expressing the mycobacterial che9 phage recombinase under control of an isovaleronitrile inducible promoter (pNIT). Step (4), successful recombination results in a chromosomal deletion of *lprG-rv1410c* with replacement by the chloramphenicol/hygromycin cassette. The recombinase plasmid is cured by counter-selection on 10% sucrose.
(PDF)

S2 Fig (Related to Fig 1) Confirmation of TAG identity by collision induced dissociation (CID) mass spectrometry. Lipid extract from Mut2 was prepared for collisional MS as described in Materials and Methods. (A) CID for *m/z* 964.93 (TAG 58:0) in Fig 1C. (B) CID for *m/z* 824.77 (TAG 48:0) and deduced fragmentation showing observed diacylglyceride units (*m/z* 703.66 and *m/z* 551.50). (C) CID for *m/z* 976.72 (TAG 59:1) and deduced fragmentation showing observed diacylglyceride units (*m/z* 551.50).
(PDF)

S3 Fig (Related to Fig 1) Levels of selected lipids in the mycobacterial lipidome across strains as detected by HPLC/MS. Lipid extract from the indicated strains (Fig 1A) was purified and analyzed as described in the Materials and Methods and S1 Methods. (A) Relative intensity of non-TAG lipids detected in Mut1, Mut2, and WT mycobacteria. (B) Relative intensity of non-TAG lipids detected in Comp2, Mut2, and WT mycobacteria. Two-way ANOVA with Bonferroni post-test (Mut1, Mut2 vs. WT) and (Comp2, WT vs. Mut2), * p < 0.05, ** p < 0.01, *** p<0.001. Cardiolipin, CL (*m/z* 1423.04); monoacylated phosphatidyl-*myo*-inositol dimannoside, PIM_1_Ac_2_ (*m/z* 1451.91); phosphatidylethanolamine, PE (*m/z* 734.57); diacylglycerol, DAG (*m/z* 612.55); phosphatidylglycerol, PG (*m/z* 766.55); phosphatidylinositol, PI (*m/z* 870.60); diacyltrehalose, DAT (*m/z* 976.76); trehalose monomycolate, TMM (*m/z* 1595.43).
(PDF)

S4 Fig (Related to Fig 2) A schematic for the vesicle-based assay used to measure TAG transfer activity. Donor vesicles contain a concentration of NBD fluorophore-labeled TAG that results in partial self-quenching of the NBD fluorescence. In the presence of excess acceptor vesicles, which lack NBD-TAG, LprG extracts and binds NBD-TAG from acceptor vesicles and deposits it into donor vesicles. This activity is observed as an increase in fluorescence.
(PDF)

S5 Fig (Related to Fig 3) Western blot for native LprG shows overexpression of the LprG-Rv1410 operon. *Mtb* lysates were prepared from late logarithmic phase cells in standard 7H9 media (OADC, 0.2%glycerol, 0.05% Tween 80) then baked at 80°C for 2 hours in SDS Laemelli buffer. Culture medium was filtered twice (0.2 micron, Millipore). Protein concentration was determined for cell lysates using a non-interfering (NI) protein assay (Biosciences). Lane 1: H37Rv (WT, 12 μg/μL); Lane 2 WT culture medium; Lane 3: *ΔlprG-rv1410c* (Mut2, 12μg/μL); Lane 4: Mut2 culture medium; Lane 5: *ΔlprG-rv1410c* L5::*lprG-rv1410c* (Comp2, 12.5 μg/μL); Lane 6: Comp2 culture medium; Lane 7: WT L5::*lprG-rv1410c* (OE, 10.6 μg/μL). Lane 8: OE culture medium. Equal loading by volume for all lanes. Primary mouse monoclonal anti-LprG 1:100 (US Biologics); secondary goat anti-mouse HRP 1:10,000.
(PDF)

S6 Fig (Related to Fig 3) TAG counts increase in culture medium with overexpression of LprG-Rv1410 over a range of experimental conditions. A) Counts of TAG events from 5 μg of culture medium lipid normalized to 10,000 matching TAG events in 2.5 μg of total cell lipids within a single MS analysis (OD 0.8 +/- 0.2) from OE, WT, Mut2, and Comp2 strains. B) Normalized ion counts for control lipids detected in culture medium from (A). PDIM (*m/z* 1385.43), diacylglycerol, DAG (*m/z* 612.55), monoacylated phosphatidyl-*myo*-inositol dimannoside, PIM_1_Ac_2_ (*m/z* 1451.91), cardiolipin, CL (*m/z* 1423.04), phosphatidylethanolamine, PE (*m/z* 734.57). C) Counts of TAG events in 10μg culture medium normalized to 100,000 matching TAG events in 5 μg total cell lipid (OD 0.7 +/- 0.2) from OE, WT, Mut2, and Comp2 strains. (A,B,C) OE vs. WT ‡, OE vs. Mut2 †, OE vs. Comp2 * p<0.05, ** p<0.01, ***p<0.001; 2-way ANOVA with Bonferroni post-test correction. D) Counts of all TAG events (48:0–60:0) from 10 μg culture medium normalized to 100,000 matching TAG events in 5 μg total cell lipid from cultures harvested over three consecutive days: late log (OD 0.5–0.9), early stationary (OD 1.0–1.2), and late stationary (OD 1.5–2.0). OE vs. WT ‡, OE vs. Mut2 †, OE vs. Comp2 * p<0.05, ** p<0.01, ***p<0.001. One-way ANOVA with Bonferroni post-test correction. TAG on average is higher in culture medium with overexpression but decreases over time (D). Normalization was performed to account for the indicated variation in OD at time of collection.
(PDF)

S7 Fig (Related to Figs 4 and 5) Complementation of LprG-Rv1410 operon restores virulence in C57/Bl6 mice. A) Composite graph of cfu recovered from the lungs of mice in two competition experiments: Mut1:WT (3:1) and Comp1:WT (3:1), as performed in Materials and Methods. B) Cfu recovered from the spleens of mice (five per group) receiving single strain IV tail vein infections with 1x10^6^ cfu of WT, Mut1, or Comp1. Cfu = colony-forming units. Mut1 = mutant 1, WT = wild-type (Fig 1A), Comp1 = *rv1410c*::*Tn* + pMV762 *lprG-rv1410c*.
(PDF)

S8 Fig (Related to Fig 5) Mut2 infected SCID mice show comparable gross and microscopic pathological change to WT and Comp2 infected mice at time of death. SCID mice were infected by aerosol with 50 colony-forming units (cfu) of WT, Mut2, or Comp2 (Fig 1A) as described in the Materials and Methods. A) Gross images of formalin-fixed heart, spleen, lung, and kidney from a representative Mut2- infected SCID mouse at termination of survival experiment. Tan lesions viewed macroscopically represent macrophage and bacteria filled aggregates. One such lesion in the lung highlighted with a red box corresponds to histopathology as seen in (B). B) Microscopic image of formalin-fixed, paraffin embedded lung showing macrophage aggregates containing numerous acid-fast bacilli (upper, 200x magnification; inset, 600x magnification). Ziehl-Neelsen acid fast staining with methylene blue counterstain. On average 2x10^6^ cfu were recovered from Mut2 infected SCID lungs at 6 months post-infection. C) Spleen colony-forming units (cfu) recovered from WT, Mut2, and Comp2 infected SCID mice at time of sacrifice/death. Mut2 infected spleens contained on average 2.25x10^6^ cfu.
(PDF)

S9 Fig (Related to Fig 6) Vitamin B12 supplementation does not rescue growth defect of LprG-Rv1410 mutant in propionate. The indicated strains were cultured in 10 mM propionate as described in the materials and methods with or without the addition of +/- 0.5 μg/mL vitamin B12 (VitB12; dashed lines) at 37°C with shaking. Growth measured by OD 600, mean +/- standard deviation.
(PDF)

S10 Fig (Related to Fig 6) Addition of glycerol decreases susceptibility of the LprG-Rv1410 mutant to tetrahydrolipostatin. MIC was determined as described in the Supplementary Materials and Methods. The MIC for WT, Mut2, and Comp2 was 62.5–125μg/mL in 7H9+ OADC (Sigma).
(PDF)

S1 Table (Related to Figs 4 and 5) Mouse strains used for competition and survival experiments. (PDF)

S2 Table (Related to Fig 6) Effect of Carbon source restriction of growth rate of *Mtb in vitro*. (PDF)
