# Supplementary material for: Mycobacterial Metabolic Syndrome: LprG and Rv1410 Regulate Triacylglyceride Levels, Growth Rate and Virulence in Mycobacterium tuberculosis
Source: PLoS Pathog. 2016 Jan 11;12(1):e1005351. doi: 10.1371/journal.ppat.1005351 (PMC4709180; doi:10.1371/journal.ppat.1005351)
Supplement: S1 Methods — (PDF) [file ppat.1005351.s001.pdf]

S1. Supplemental Materials and Methods

Genetic Manipulation of *Mtb*, related to Figure 1, Figure S1. Mycobacterial phage-mediated recombineering was employed to create the double knockout of the *rv1411c-1410c* (LprG-Rv1410) operon based on methodology previously described (1). A cosmid library of mycobacterial genes was used to isolate the genomic template for the *lprG-rv1410c* operon. Cosmid 2_33 contains roughly 30kb of genomic DNA from *Mtb* and was confirmed to contain the genes *rv1411c-rv1410c* (LprG-Rv1410) by PCR using primers AJM1F and AJM1R. The cosmid was amplified, purified, and transformed into *E.coli DH5α* previously transformed with a temperature sensitive, arabinose inducible (pAraBAD), tetracycline-marked lambda-red recombinase (pkd119) (2). Recombination is suppressed in the presence of 1% glucose therefore culture of the pkd119 strain required maintenance in media supplemented with glucose and tetracycline. Primers with 50 bp of homology to genomic sequence upstream to *lprG* and downstream of *rv1410c* were designed to amplify a chloramphenicol/hygromycin resistance cassette. The resultant linear PCR product was transformed into the E.coli pkd 119 strain containing the 2_33 cosmid. Allelic exchange resulted in the creation of a recombinant cosmid containing the resistance cassette flanked by genomic sequence upstream and downstream of *lprG-rv1410c*.

Recombinant cosmids were amplified and purified and used as a template to generate the *Mtb* recombineering construct containing 500bp of genomic homology upstream and downstream of the *lprG-rv1410c* operon on either side of the chloramphenicol/hygromycin resistance cassette. For selection in *E.coli* chloramphenicol (15μg/mL) was used; hygromycin (50μg/mL) in *Mtb.* The linear

recombineering PCR construct was transformed into a confirmed PDIM positive mycobacterial *H37Rv* strain that had previously been transformed with mycobacterial Che9c phage recombinase -expressing pNIT::recET-sacB-kan (3) that is inducible with isovaleronitrile (IVN). Competent, recombinase-expressing mycobacteria are generated as follows: Cells are grown to OD 0.8. Cells are then incubated for 8 hours with 1mM isovaleronitrile (Sigma-Aldrich 308528) after which 2M glycine is added and cells incubated overnight. Cells are washed with 10% glycerol and the 3kb linear PCR product containing 500 bp of chromosomal homology upstream and downstream of the LprG-Rv1410 genetic locus flanking the chloramphenicol/hygromycin resistance cassette is electroporated into mycobacterial cells as previously described (4). Recombinants were selected on hygromycin (50µg/mL) and PCR confirmed (AJM15F,R; AJM16F,R). The recombinase plasmid was cured by counter-selection on 10% sucrose in the absence of kanamycin and confirmed by patching. The knock-out was confirmed by whole genome sequencing (Fig S1). For generation of the *lprG-rv1410c* complemented strains (Comp2, Mut2 +LprG, and Mut2+ Rv1410) and overexpression strain (OE), *H37Rv* (WT) and Δ*lprG-rv1410c* (Mut2) were transformed with Gateway ® (Thermo Fisher Scientific) destination vectors containing the *lprG, rv1410c, or lprG-rv1410c* genes flanked by 500bp sequence homolog to the mycobacterial chromosomal L5 site. Transformants were selected on zeocin 20µg/mL. Complemented strains and OE were maintained with zeocin 20µg/mL in broth culture; no antibiotic used for recovery on plates.

Primers used in genetic manipulation of *Mtb*.

ajm1F	Recombineering Step 1: confirm gene presence on cosmid 2_33	ATGCGGACCCCCAGACGCCA
ajm1R	Recombineering Step 1: confirm gene presence on cosmid 2_33	TTAGAGCGGCTCCACTTGGG
ajm2F	E.coli Recombineering for LprG-Rv1410 KO Step 2: 50bp homology flanking resistance cassette	ACCCCACTCGCCGACCGGCCAACTCACAGACACCCTCTACGATGCAGGGT GGCCGACCAGCCCGT
ajm2R	E.coli Recombineering for LprG-Rv1410 KO Step 2: 50bp homology flanking resistance cassette	GACAGCGTCGAAAAGGTCGGACCGGATCTGTTGCTGAGCTTGGTGGCTCGGCCAGCTAGAGGGGC
ajm3F	E.coli Recombineering for LprG-Rv1410 KO Step 3: PCR *Mtb* recombineering construct using recombineered cosmid	GAGCCACCGAAGCCGGCATC
ajm3R	E.coli Recombineering for LprG-Rv1410 KO Step 3: PCR *Mtb* recombineering construct using recombineered cosmid	GCGCCGCCGCCGACGGCTCC
ajm8F	Hygromycin_F	AACTGCATCTCAACGCCTTC
ajm8R	Chloramphenicol_R	ATCACA AACGGCATGATGAA
ajm15F	outside 500bp flank--sequencing	CCGCATTAACCGCTC
ajm15R	Hygromycin sequencing	CAGGCGTTGACGATGAC
ajm16Fa	Chloramphenicol--sequencing	TTCATCATGCCGTTTGTGAT
ajm16Ra	outside 500bp flank--sequencing	CTGCCGCATGTCACCT

R, Gene Pattern, and Statistical Analysis, related to Figure 1. Raw data files were converted to mzData files using MassHunter (Agilent Technologies) and processed in R using XCMS conversion (v1.24) (5) allowing peak deconvolution and alignment across samples as previously described (6). The dataset was exported into GenePattern (v2.0, Broad Institute). Using the “Multiplot Preprocess” and “Multiplot” functions, triplicate lipidomic data was used to generate “volcano” plots comparing the lipidomes of two *Mtb* strains, such as “Mut2 or Mut1” versus “WT,” or “Mut2” versus “Comp2.” A filter for changes two fold or greater at p-value <0.05 was used to identify variations in lipid features between strains using a student’s paired t-test and Hochberg multiple comparison correction (7).

Cloning of lipoprotein expression vectors, related to Figure 2. A non-acylated (NA) version of MtbLprG (NA-MtbLprG) that excludes the signal sequence and changes the acylated cysteine to a methionine was cloned from *Mtb* H37Rv genomic DNA. Site-directed mutagenesis of NA-MtbLprG was performed to achieve a V91W amino acid mutation. For expression in *E. coli*, the target gene was cloned into pET24b behind the IPTG-inducible T7-lac promoter and in frame with a TEV protease recognition site and a C-terminal His_6-tag.

Overexpression of His_6-tagged recombinant proteins, related to Figure 2. For expression in *E. coli*, BL21 (DE3) competent cells were transformed with the pET24b constructs and cultured in Luria-Bertani broth (LB); 50 μg/mL kanamycin was used for selection. When the OD_{600} was approximately 1, 0.1 mM IPTG was added to induce protein expression for 4 h at 37 °C. Bacteria were isolated by centrifugation at 5000 xg for 20 min at 4 °C. The pellet was stored at -20 °C or lysed immediately.

Purification of His_6-tagged proteins, related to Figure 2. All steps were performed at 4°C unless otherwise noted. To purify proteins from *E. coli*, cell pellets were resuspended in lysis buffer (20 mM Tris, 200 mM NaCl, 1 mM DTT, 0.2 mM EDTA, 10 mM imidazole, 10% glycerol, pH 7.4) and sonicated 5s on/off for 10 min total processing time. Nickel affinity chromatography (HisTrap FF 5mL, GE Healthcare) was used for initial purification. After injection, protein samples were washed with 5 column volume (CV) binding buffer (Buffer A, 50 mM Tris, 1 mM DTT, 10% glycerol, pH 7.4), and bound proteins were eluted with a 0-50% gradient of elution buffer (Buffer A with 1M imidazole) over 20 CV. All target proteins eluted at an imidazole concentration of approximately 100 mM and were analyzed for purity by SDS-PAGE. If contaminating proteins co-eluted, a second purification step by anion exchange chromatography (HiTrap Q HP 5 mL, GE

Healthcare) was performed. The protein was eluted with a gradient of 0-100% high salt buffer (Buffer A with 1M NaCl) over 20 CV. The pH of the buffer was adjusted to at least 1 unit higher than the theoretical isoelectric point (pI) of the target protein. All of the proteins were purified in a final step by size exclusion (HiPrep 16/60 120 mL, GE Healthcare). The mobile phase was buffer A without glycerol. Purified proteins were verified by tryptic digest and mass spectrometry (MS), flash frozen in liquid nitrogen and stored at -80 °C in Buffer A. Using the theoretical extinction coefficient, proteins' concentrations were estimated by A_{280}. All of the non-acylated proteins (NA-MtbLprG, V91W NA-MtbLprG, NA-MtbLprA, NA-MsmegLprG and A91W NA-MsmegLprG) were stably expressed in *E. coli* and/or in *M. smegmatis*; purified and verified by MS as described above.

Minimal inhibitory Concentration (MIC) of Tetrahydrolipostatin, related to Figure 6 and Figure S10. Mycobacteria were grown to log phase and diluted back to OD 0.006. Serial dilutions of tetrahydrolipostatin (THL) (Sigma) were made in 96 well plates in either 7H9 with OADC and 0.2% glycerol (Fig S10) or Sauton's 0.8% glycerol (data not shown). Mycobacteria were added to wells (total volume 200μL) and allowed to grow for 6 days at 37°C with shaking. Resazurin 0.02% was added on the 6th day at 10μL per well and cells were incubated overnight as above. Color change from pink to purple/blue indicated growth inhibition (pink positive control). MIC for WT, Mut2, and Comp2 was 62.5-125 μg/mL in 7H9+OADC 0.2% glycerol; 4-8 μg/mL in Sauton's 0.8% glycerol.

Histopathological analysis of mouse tissue, related to Figure 5*.* Spleen, liver, kidney, heart and lung were collected in 10% neutral buffered formalin (NBF), fixed for 24 hours in 10% NBF and stored at 4°C in 70% ethanol. Lungs were inflated with 10%

NBF prior to fixation. Fixed organs were embedded in paraffin, and lung sections were stained with hematoxylin-eosin, or Ziehl Neelsen Method acid-fast staining, and evaluated by light microscopy.

REFERENCES

1. Murphy KC. Targeted chromosomal gene knockout using PCR fragments. Methods Mol Biol. 2011;765:27–42.

2. Murphy KC. The lambda Gam protein inhibits RecBCD binding to dsDNA ends. J Mol Biol. 2007 Aug 3;371(1):19–24.

3. Wei J-R, Krishnamoorthy V, Murphy K, Kim J-H, Schnappinger D, Alber T, et al. Depletion of antibiotic targets has widely varying effects on growth. Proc Natl Acad Sci USA. 2011 Mar 8;108(10):4176–81.

4. Hatfull GF, Jacobs WR Jr. Molecular Genetics of Mycobacteria. ASM Press; 2014. 1 p.

5. Smith CA, Want EJ, O'Maille G, Abagyan R, Siuzdak G. XCMS: Processing Mass Spectrometry Data for Metabolite Profiling Using Nonlinear Peak Alignment, Matching, and Identification. Anal Chem. 2006 Feb;78(3):779–87.

6. Layre E, Sweet L, Hong S, Madigan CA, Desjardins D, Young DC, et al. A Comparative Lipidomics Platform for Chemotaxonomic Analysis of Mycobacterium tuberculosis. Chem Biol [Internet]. Elsevier Ltd; 2011 Dec 23;18(12):1537–49. Available from: http://linkinghub.elsevier.com/retrieve/pii/S107455211100398X

7. Benjamini Y, Hochberg Y. Controlling the False Discovery Rate: A Practical and Powerful Approach to Multiple Testing. Journal of the Royal Statistical Society [Internet]. Series B. 1995 Mar 8;57(1):289–300. Available from: http://www.jstor.org/stable/2346101
