# Supplementary material for: Mycobacterial Metabolic Syndrome: LprG and Rv1410 Regulate Triacylglyceride Levels, Growth Rate and Virulence in Mycobacterium tuberculosis
Source: PLoS Pathog. 2016 Jan 11;12(1):e1005351. doi: 10.1371/journal.ppat.1005351 (PMC4709180; doi:10.1371/journal.ppat.1005351)
Supplement: S1 Fig — The cosmid 2_33 containing the lprG-rv1410c genes was used as a template to amplify a linear PCR product (ajm2F, ajm2R) with 50 basepair (bp) homology upstream and down stream of the operon flanking a chloramphenicol/hygromycin (Chlor/Hyg) resistance cassette. Step (1) Recombineering was performed in the E.coli pkD119 strain containing a lambda-red recombinase plasmid to exchange the Chlor/Hyg cassette with the lprG-rv1410c genetic locus on the 2_33 cosmid. Step (2) the recombinant cosmid is used as a template to generate a linear PCR product (ajm3F, ajm3R) with 500 bp chromosomal homology upstream and downstream of the lprG-rv1410c operon (replaced by Chlor/Hyg). Step (3) the 3kb linear PCR product is transformed into confirmed PDIM positive Mtb (H37Rv) previously transformed with pRecET, expressing the mycobacterial che9 phage recombinase under control of an isovaleronitrile inducible promoter (pNIT). Step (4), successful recombination results in a chromosomal deletion of lprG-rv1410c with replacement by the chloramphenicol/hygromycin cassette. The recombinase plasmid is cured by counter-selection on 10% sucrose. (PDF) [file ppat.1005351.s002.pdf]

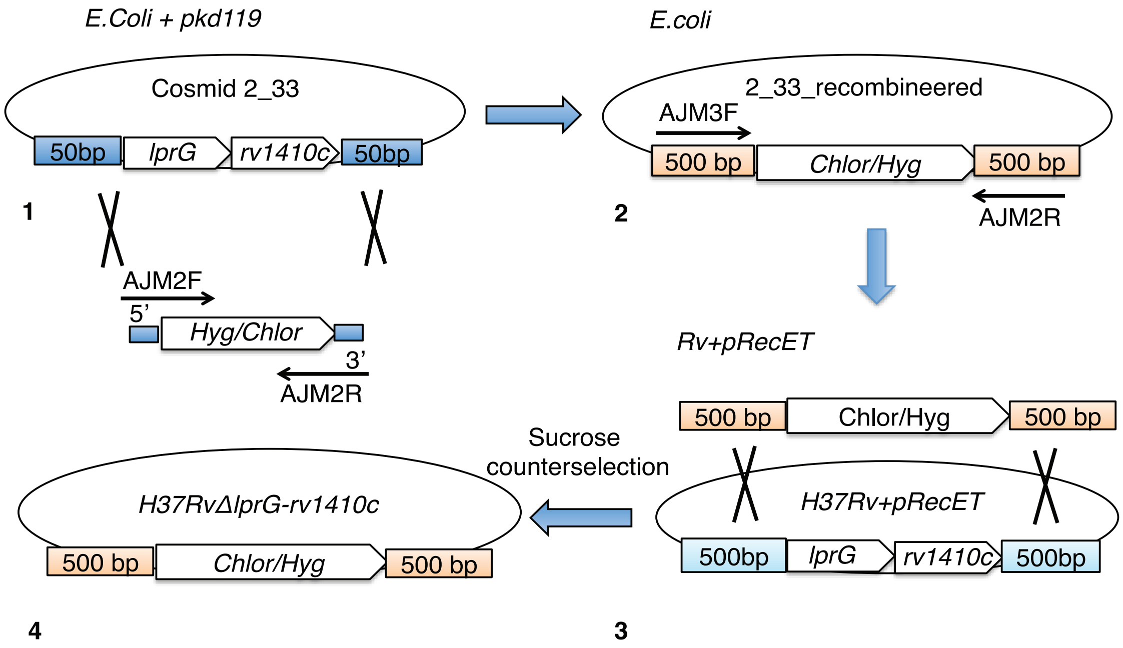

Figure S1, related to Figure 1 and supplemental experimental procedures. Genetic manipulation of *Mtb* to generate LprG-Rv1410 double knockout. The cosmid 2_33 containing the *lprG-rv1410c* genes was used as a template to amplify a linear PCR product (ajm2F, ajm2R) with 50 basepair (bp) homology upstream and down stream of the operon flanking a chloramphenicol/hygromycin (Chlor/Hyg) resistance cassette. Step (1) Recombineering was performed in the *E.coli* pkD119 strain containing a lambda-red recombinase plasmid to exchange the Chlor/Hyg cassette with the *lprG-rv1410c* genetic locus on the 2_33 cosmid. Step (2) the recombinant cosmid is used as a template to generate a linear PCR product (ajm3F, ajm3R) with 500 bp chromosomal homology upstream and downstream of the *lprG-rv1410c* operon (replaced by Chlor/Hyg). Step (3) the 3kb linear PCR product is transformed into confirmed PDIM positive *Mtb (H37Rv)* previously transformed with pRecET, expressing the mycobacterial che9 phage recombinase under control of an isovaleronitrile inducible promoter (pNIT). Step (4), successful recombination results in a chromosomal deletion of *lprG-rv1410c* with replacement by the chloramphenicol/hygromycin cassette. The recombinase plasmid is cured by counter-selection on 10% sucrose.
