# Supplementary material for: Mycobacterial Metabolic Syndrome: LprG and Rv1410 Regulate Triacylglyceride Levels, Growth Rate and Virulence in Mycobacterium tuberculosis
Source: PLoS Pathog. 2016 Jan 11;12(1):e1005351. doi: 10.1371/journal.ppat.1005351 (PMC4709180; doi:10.1371/journal.ppat.1005351)
Supplement: S2 Fig — Lipid extract from Mut2 was prepared for collisional MS as described in Materials and Methods. (A) CID for m/z 964.93 (TAG 58:0) in Fig 1C. (B) CID for m/z 824.77 (TAG 48:0) and deduced fragmentation showing observed diacylglyceride units (m/z 703.66 and m/z 551.50). (C) CID for m/z 976.72 (TAG 59:1) and deduced fragmentation showing observed diacylglyceride units (m/z 551.50). (PDF) [file ppat.1005351.s003.pdf]

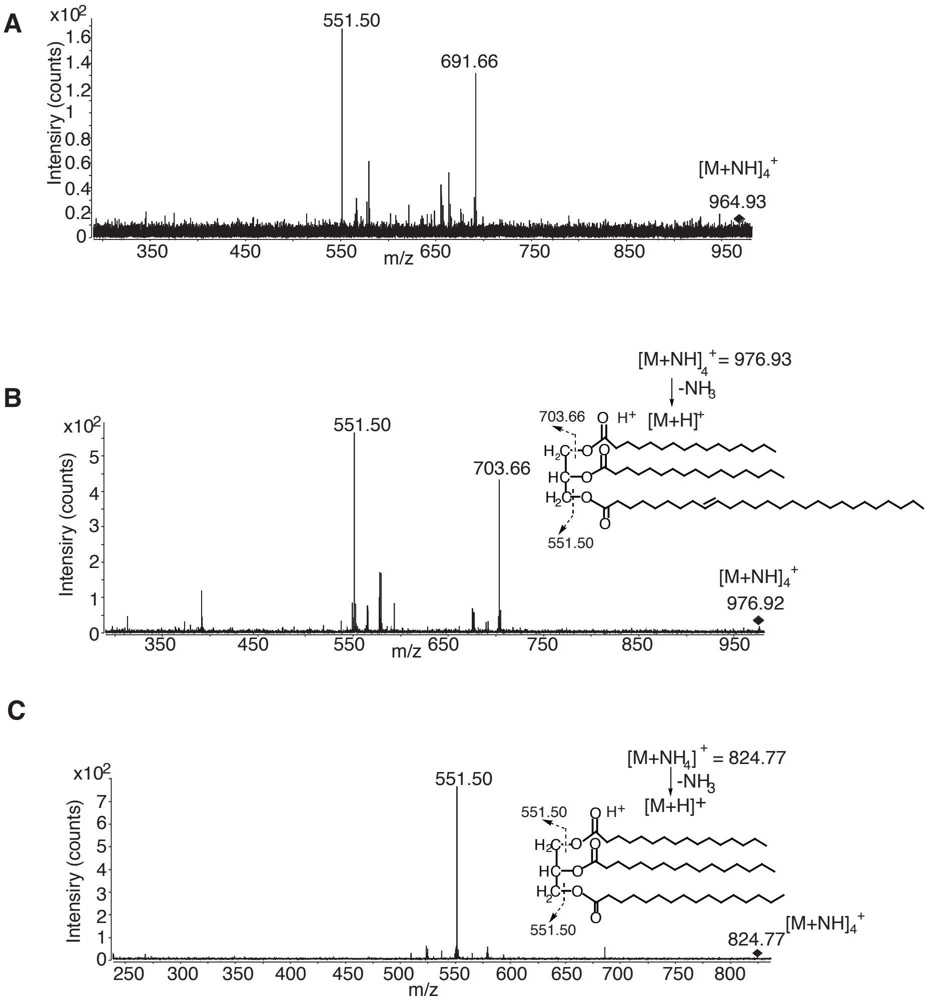

Figure S2, related to Figure 1. Confirmation of TAG identity by collision induced dissociation (CID) mass spectrometry. Lipid extract from Mut2 was prepared for collisional MS as described in Materials and Methods. (A) CID for *m/z* 964.93 (TAG 58:0) in Figure 1C. (B) CID for *m/z* 824.77 (TAG 48:0) and deduced fragmentation showing observed diacylglyceride units (*m/z* 703.66 and *m/z* 551.50). (C) CID for *m/z* 976.72 (TAG 59:1) and deduced fragmentation showing observed diacylglyceride units (*m/z* 551.50).
