# Supplementary material for: Mycobacterial Metabolic Syndrome: LprG and Rv1410 Regulate Triacylglyceride Levels, Growth Rate and Virulence in Mycobacterium tuberculosis
Source: PLoS Pathog. 2016 Jan 11;12(1):e1005351. doi: 10.1371/journal.ppat.1005351 (PMC4709180; doi:10.1371/journal.ppat.1005351)
Supplement: S3 Fig — Lipid extract from the indicated strains (Fig 1A) was purified and analyzed as described in the Materials and Methods and S1 Methods. (A) Relative intensity of non-TAG lipids detected in Mut1, Mut2, and WT mycobacteria. (B) Relative intensity of non-TAG lipids detected in Comp2, Mut2, and WT mycobacteria. Two-way ANOVA with Bonferroni post-test (Mut1, Mut2 vs. WT) and (Comp2, WT vs. Mut2), * p < 0.05, ** p < 0.01, *** p<0.001. Cardiolipin, CL (m/z 1423.04); monoacylated phosphatidyl-myo-inositol dimannoside, PIM1Ac2 (m/z 1451.91); phosphatidylethanolamine, PE (m/z 734.57); diacylglycerol, DAG (m/z 612.55); phosphatidylglycerol, PG (m/z 766.55); phosphatidylinositol, PI (m/z 870.60); diacyltrehalose, DAT (m/z 976.76); trehalose monomycolate, TMM (m/z 1595.43). (PDF) [file ppat.1005351.s004.pdf]

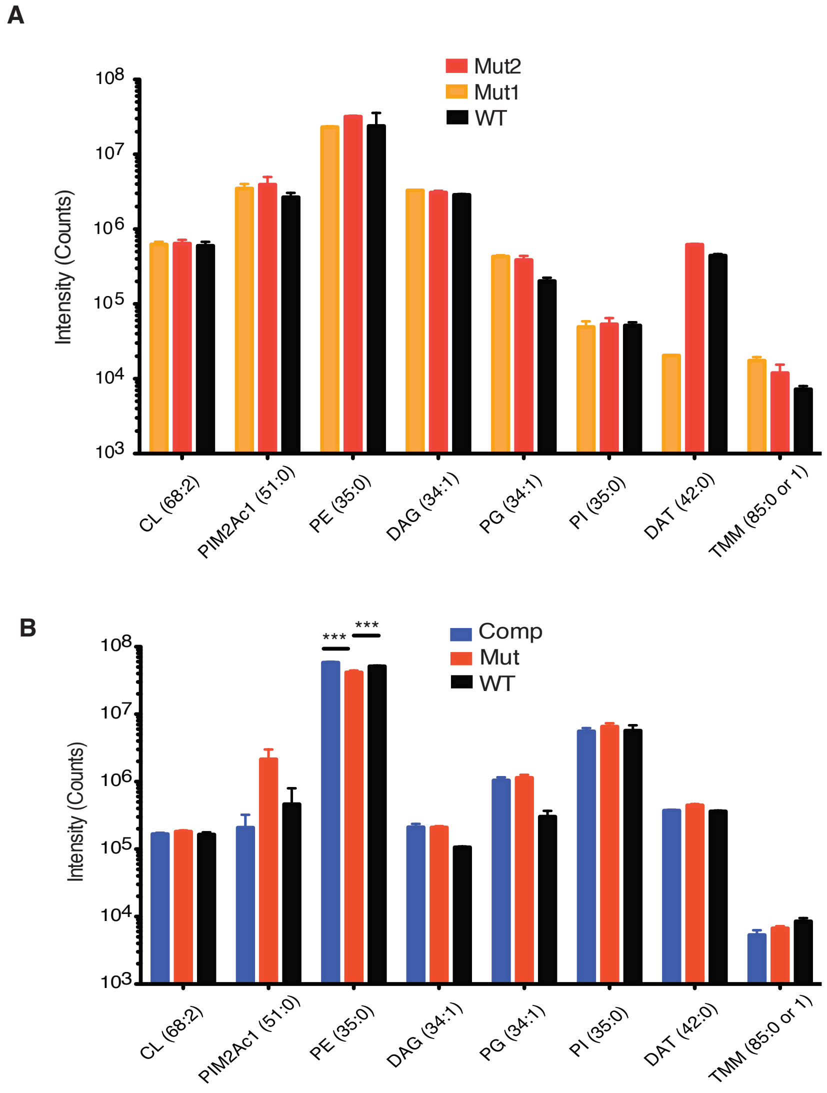

Figure S3, related to Figure 1. Levels of selected lipids in the mycobacterial lipidome across strains as detected by HPLC/MS. Lipid extract from the indicated strains (Fig 1A) was purified and analyzed as described in the Materials and Methods and S1 Methods. (A) Relative intensity of non-TAG lipids detected in Mut1, Mut2, and WT mycobacteria. (B) Relative intensity of non-TAG lipids detected in Comp2, Mut2, and WT mycobacteria. Two-way ANOVA with Bonferroni post-test (Mut1, Mut2 vs. WT) and (Comp2, WT vs. Mut2), * $p < 0.05$, ** $p < 0.01$, *** $p<0.001$. Cardiolipin, CL (*m/z* 1423.04); monoacylated phosphatidyl-*myo*-inositol dimannoside, PIM_1Ac_2 (*m/z* 1451.91); phosphatidylethanolamine, PE (*m/z* 734.57); diacylglycerol, DAG (*m/z* 612.55); phosphatidylglycerol, PG (*m/z* 766.55); phosphatidylinositol, PI (*m/z* 870.60); diacyltrehalose, DAT (*m/z* 976.76); trehalose monomycolate, TMM (*m/z* 1595.43).
