# Supplementary material for: Mycobacterial Metabolic Syndrome: LprG and Rv1410 Regulate Triacylglyceride Levels, Growth Rate and Virulence in Mycobacterium tuberculosis
Source: PLoS Pathog. 2016 Jan 11;12(1):e1005351. doi: 10.1371/journal.ppat.1005351 (PMC4709180; doi:10.1371/journal.ppat.1005351)
Supplement: S4 Fig — Donor vesicles contain a concentration of NBD fluorophore-labeled TAG that results in partial self-quenching of the NBD fluorescence. In the presence of excess acceptor vesicles, which lack NBD-TAG, LprG extracts and binds NBD-TAG from acceptor vesicles and deposits it into donor vesicles. This activity is observed as an increase in fluorescence. (PDF) [file ppat.1005351.s005.pdf]

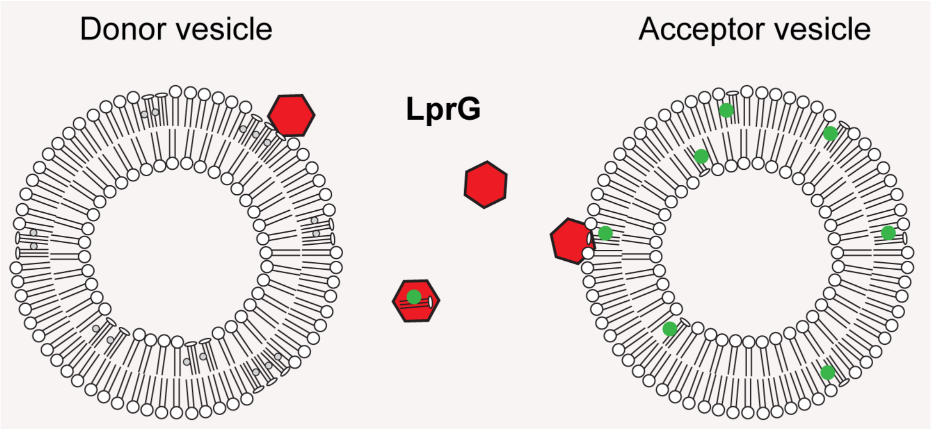

Figure S4, related to Figure 2. A schematic for the vesicle-based assay used to measure TAG transfer activity. Donor vesicles contain a concentration of NBD fluorophore-labeled TAG that results in partial self-quenching of the NBD fluorescence. In the presence of excess acceptor vesicles, which lack NBD-TAG, LprG extracts and binds NBD-TAG from acceptor vesicles and deposits it into donor vesicles. This activity is observed as an increase in fluorescence.
