# Supplementary material for: Mycobacterial Metabolic Syndrome: LprG and Rv1410 Regulate Triacylglyceride Levels, Growth Rate and Virulence in Mycobacterium tuberculosis
Source: PLoS Pathog. 2016 Jan 11;12(1):e1005351. doi: 10.1371/journal.ppat.1005351 (PMC4709180; doi:10.1371/journal.ppat.1005351)
Supplement: S5 Fig — Mtb lysates were prepared from late logarithmic phase cells in standard 7H9 media (OADC, 0.2%glycerol, 0.05% Tween 80) then baked at 80°C for 2 hours in SDS Laemelli buffer. Culture medium was filtered twice (0.2 micron, Millipore). Protein concentration was determined for cell lysates using a non-interfering (NI) protein assay (Biosciences). Lane 1: H37Rv (WT, 12 μg/μL); Lane 2 WT culture medium; Lane 3: ΔlprG-rv1410c (Mut2, 12μg/μL); Lane 4: Mut2 culture medium; Lane 5: ΔlprG-rv1410c L5::lprG-rv1410c (Comp2, 12.5 μg/μL); Lane 6: Comp2 culture medium; Lane 7: WT L5::lprG-rv1410c (OE, 10.6 μg/μL). Lane 8: OE culture medium. Equal loading by volume for all lanes. Primary mouse monoclonal anti-LprG 1:100 (US Biologics); secondary goat anti-mouse HRP 1:10,000. (PDF) [file ppat.1005351.s006.pdf]

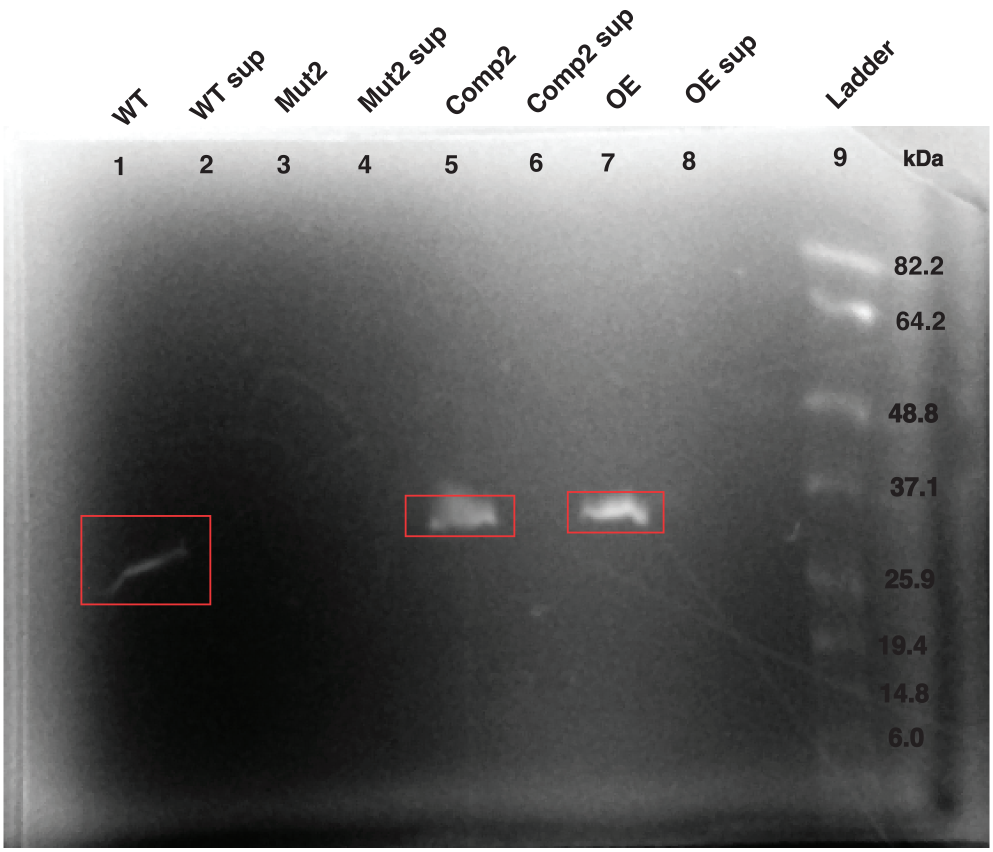

Figure S5 (related to Figure 3). Western Blot for native LprG shows overexpression of the LprG-Rv1410 operon. Mtb lysates were prepared from late logarithmic phase cells in standard 7H9 media (OADC, 0.2%glycerol, 0.05% Tween 80) then baked at 80°C for 2 hours in SDS—Laemelli buffer. Culture medium was filtered twice (0.2 micron, Millipore). Protein concentration was determined for cell lysates using a non-interfering (NI) protein assay (Biosciences). Lane 1: H37Rv (WT, 12 µg/µL); Lane 2 WT culture medium; Lane 3: *ΔlprG-rv1410c* (Mut2, 12µg/µL); Lane 4: Mut2 culture medium; Lane 5: *ΔlprG-rv1410c* L5*::lprG-rv1410c* (Comp2, 12.5 µg/µL); Lane 6: Comp2 culture medium; Lane 7: WT L5*::lprG-rv1410c* (OE, 10.6 µg/µL). Lane 8: OE culture medium. Equal loading by volume for all lanes. Primary mouse monoclonal anti-LprG 1:100 (US Biologics); secondary goat anti-mouse HRP 1:10,000.
