# Supplementary material for: Mycobacterial Metabolic Syndrome: LprG and Rv1410 Regulate Triacylglyceride Levels, Growth Rate and Virulence in Mycobacterium tuberculosis
Source: PLoS Pathog. 2016 Jan 11;12(1):e1005351. doi: 10.1371/journal.ppat.1005351 (PMC4709180; doi:10.1371/journal.ppat.1005351)
Supplement: S6 Fig — A) Counts of TAG events from 5 μg of culture medium lipid normalized to 10,000 matching TAG events in 2.5 μg of total cell lipids within a single MS analysis (OD 0.8 +/- 0.2) from OE, WT, Mut2, and Comp2 strains. B) Normalized ion counts for control lipids detected in culture medium from (A). PDIM (m/z 1385.43), diacylglycerol, DAG (m/z 612.55), monoacylated phosphatidyl-myo-inositol dimannoside, PIM1Ac2 (m/z 1451.91), cardiolipin, CL (m/z 1423.04), phosphatidylethanolamine, PE (m/z 734.57). C) Counts of TAG events in 10μg culture medium normalized to 100,000 matching TAG events in 5 μg total cell lipid (OD 0.7 +/- 0.2) from OE, WT, Mut2, and Comp2 strains. (A,B,C) OE vs. WT ‡, OE vs. Mut2 †, OE vs. Comp2 * p<0.05, ** p<0.01, ***p<0.001; 2-way ANOVA with Bonferroni post-test correction. D) Counts of all TAG events (48:0–60:0) from 10 μg culture medium normalized to 100,000 matching TAG events in 5 μg total cell lipid from cultures harvested over three consecutive days: late log (OD 0.5–0.9), early stationary (OD 1.0–1.2), and late stationary (OD 1.5–2.0). OE vs. WT ‡, OE vs. Mut2 †, OE vs. Comp2 * p<0.05, ** p<0.01, ***p<0.001. One-way ANOVA with Bonferroni post-test correction. TAG on average is higher in culture medium with overexpression but decreases over time (D). Normalization was performed to account for the indicated variation in OD at time of collection. (PDF) [file ppat.1005351.s007.pdf]

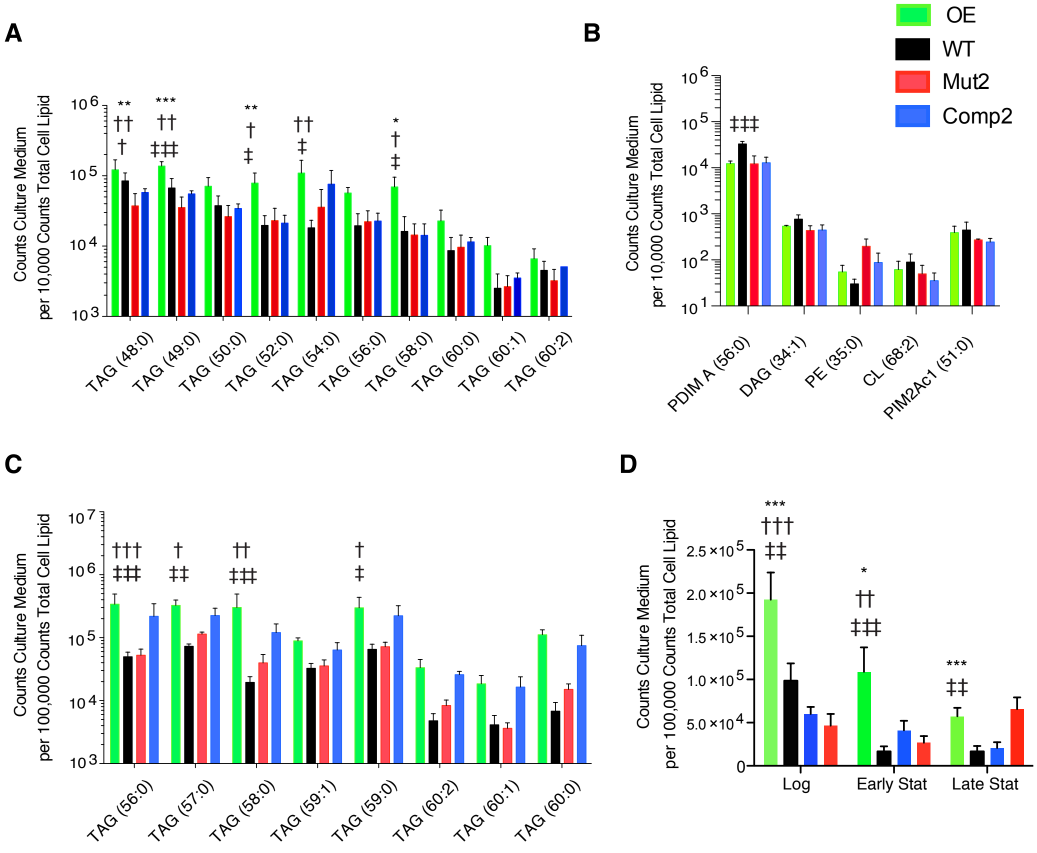

Figure S6 (related to Figure 3). TAG counts increase in culture medium with overexpression of LprG-Rv1410 over a range of experimental conditions. A) Counts of TAG events from 5 μg of culture medium lipid normalized to 10,000 matching TAG events in 2.5 μg of total cell lipids within a single MS analysis (OD 0.8 +/- 0.2) from OE, WT, Mut2, and Comp2 strains. B) Normalized ion counts for control lipids detected in culture medium from (A). PDIM (*m/z* 1385.43), diacylglycerol, DAG (*m/z* 612.55), monoacylated phosphatidyl-*myo*-inositol dimannoside, PIM_1Ac_2 (*m/z* 1451.91), cardiolipin, CL (*m/z* 1423.04), phosphatidylethanolamine, PE (*m/z* 734.57). C) Counts of TAG events in 10μg culture medium normalized to 100,000 matching TAG events in 5 μg total cell lipid (OD 0.7 +/- 0.2) from OE, WT, Mut2, and Comp2 strains. (A,B,C) OE vs. WT ‡, OE vs. Mut2 †, OE vs. Comp2 * p<0.05, ** p<0.01, ***p<0.001; 2-way ANOVA with Bonferroni post-test correction. D) Counts of all TAG events (48:0-60:0) from 10 μg culture medium normalized to 100,000 matching TAG events in 5 μg total cell lipid from cultures harvested over three consecutive days: late log (OD 0.5-0.9), early stationary (OD 1.0-1.2), and late stationary (OD 1.5-2.0). OE vs. WT ‡, OE vs. Mut2 †, OE vs. Comp2 * p<0.05, ** p<0.01, ***p<0.001. One-way ANOVA with Bonferroni post-test correction. TAG on average is higher in culture medium with overexpression but decreases over time (D). Normalization was performed to account for the indicated variation in OD at time of collection.
