# Supplementary material for: Mycobacterial Metabolic Syndrome: LprG and Rv1410 Regulate Triacylglyceride Levels, Growth Rate and Virulence in Mycobacterium tuberculosis
Source: PLoS Pathog. 2016 Jan 11;12(1):e1005351. doi: 10.1371/journal.ppat.1005351 (PMC4709180; doi:10.1371/journal.ppat.1005351)
Supplement: S7 Fig — A) Composite graph of cfu recovered from the lungs of mice in two competition experiments: Mut1:WT (3:1) and Comp1:WT (3:1), as performed in Materials and Methods. B) Cfu recovered from the spleens of mice (five per group) receiving single strain IV tail vein infections with 1x10^6^ cfu of WT, Mut1, or Comp1. Cfu = colony-forming units. Mut1 = mutant 1, WT = wild-type (Fig 1A), Comp1 = rv1410c::Tn + pMV762 lprG-rv1410c. (PDF) [file ppat.1005351.s008.pdf]

A

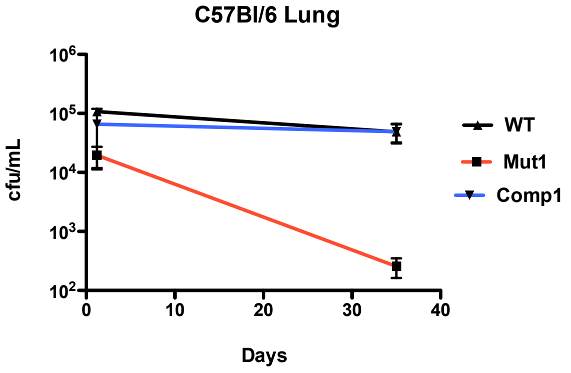

B

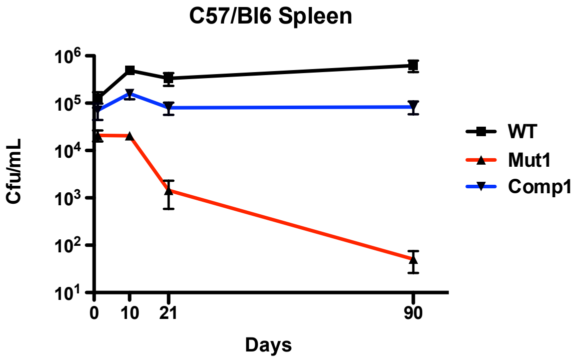

Figure S7 (related to Figure 4). Complementation of LprG-Rv1410 operon restores virulence in C57/Bl6 mice. A) Composite graph of cfu recovered from the lungs of mice in two competition experiments: Mut1:WT (3:1) and Comp1:WT (3:1), as performed in Materials and Methods. B) Cfu recovered from the spleens of mice (five per group) receiving single strain IV tail vein infections with $1x10^6$ cfu of WT, Mut1, or Comp1. Cfu=colony-forming units. Mut1=mutant 1, WT=wild-type (Figure 1A), Comp1= *rv1410c*::*Tn* + pMV762 *lprG-rv1410c.*
