# Supplementary material for: Mycobacterial Metabolic Syndrome: LprG and Rv1410 Regulate Triacylglyceride Levels, Growth Rate and Virulence in Mycobacterium tuberculosis
Source: PLoS Pathog. 2016 Jan 11;12(1):e1005351. doi: 10.1371/journal.ppat.1005351 (PMC4709180; doi:10.1371/journal.ppat.1005351)
Supplement: S8 Fig — SCID mice were infected by aerosol with 50 colony-forming units (cfu) of WT, Mut2, or Comp2 (Fig 1A) as described in the Materials and Methods. A) Gross images of formalin-fixed heart, spleen, lung, and kidney from a representative Mut2- infected SCID mouse at termination of survival experiment. Tan lesions viewed macroscopically represent macrophage and bacteria filled aggregates. One such lesion in the lung highlighted with a red box corresponds to histopathology as seen in (B). B) Microscopic image of formalin-fixed, paraffin embedded lung showing macrophage aggregates containing numerous acid-fast bacilli (upper, 200x magnification; inset, 600x magnification). Ziehl-Neelsen acid fast staining with methylene blue counterstain. On average 2x10^6^ cfu were recovered from Mut2 infected SCID lungs at 6 months post-infection. C) Spleen colony-forming units (cfu) recovered from WT, Mut2, and Comp2 infected SCID mice at time of sacrifice/death. Mut2 infected spleens contained on average 2.25x10^6^ cfu. (PDF) [file ppat.1005351.s009.pdf]

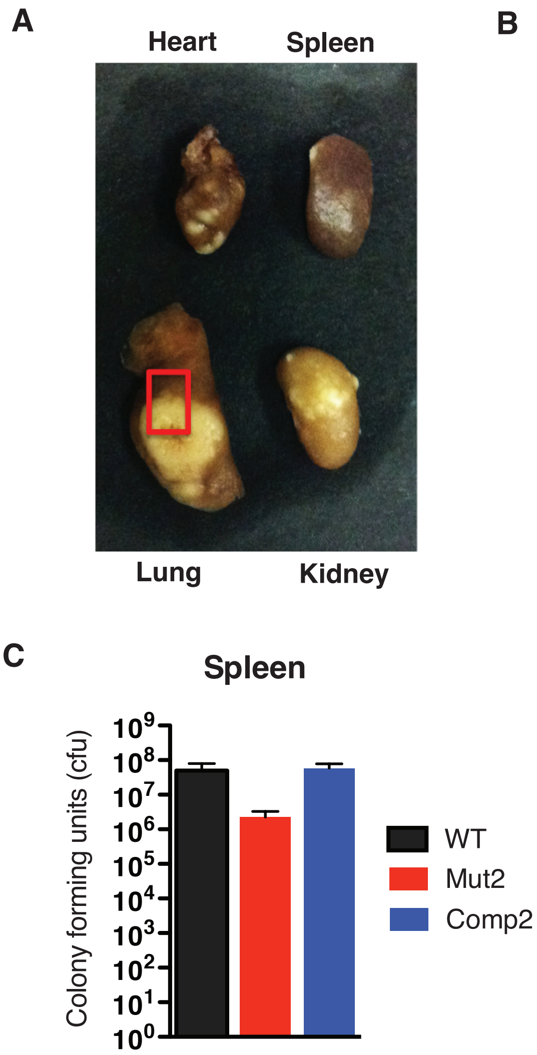

Figure S8 (related to Figure 5). Mut2 infected SCID mice show comparable gross and microscopic pathological change to WT and Comp2 infected mice at time of death. SCID mice were infected by aerosol with 50 colony-forming units (cfu) of WT, Mut2, or Comp2 (Fig 1A) as described in the Materials and Methods. A) Gross images of formalin-fixed heart, spleen, lung, and kidney from a representative Mut2-infected SCID mouse at termination of survival experiment. Tan lesions viewed macroscopically represent macrophage and bacteria filled aggregates. One such lesion in the lung highlighted with a red box corresponds to histopathology as seen in (B). B) Microscopic image of formalin-fixed, paraffin embedded lung showing macrophage aggregates containing numerous acid-fast bacilli (upper, 200x magnification; inset, 600x magnification). Ziehl-Neelsen acid fast staining with methylene blue counterstain. On average $2x10^6$ cfu were recovered from Mut2 infected SCID lungs at 6 months post-infection. C) Spleen colony-forming units (cfu) recovered from WT, Mut2, and Comp2 infected SCID mice at time of sacrifice/death. Mut2 infected spleens contained on average $2.25x10^6$ cfu.
