# Supplementary material for: Mycobacterial Metabolic Syndrome: LprG and Rv1410 Regulate Triacylglyceride Levels, Growth Rate and Virulence in Mycobacterium tuberculosis
Source: PLoS Pathog. 2016 Jan 11;12(1):e1005351. doi: 10.1371/journal.ppat.1005351 (PMC4709180; doi:10.1371/journal.ppat.1005351)
Supplement: S9 Fig — The indicated strains were cultured in 10 mM propionate as described in the materials and methods with or without the addition of +/- 0.5 μg/mL vitamin B12 (VitB12; dashed lines) at 37°C with shaking. Growth measured by OD 600, mean +/- standard deviation. (PDF) [file ppat.1005351.s010.pdf]

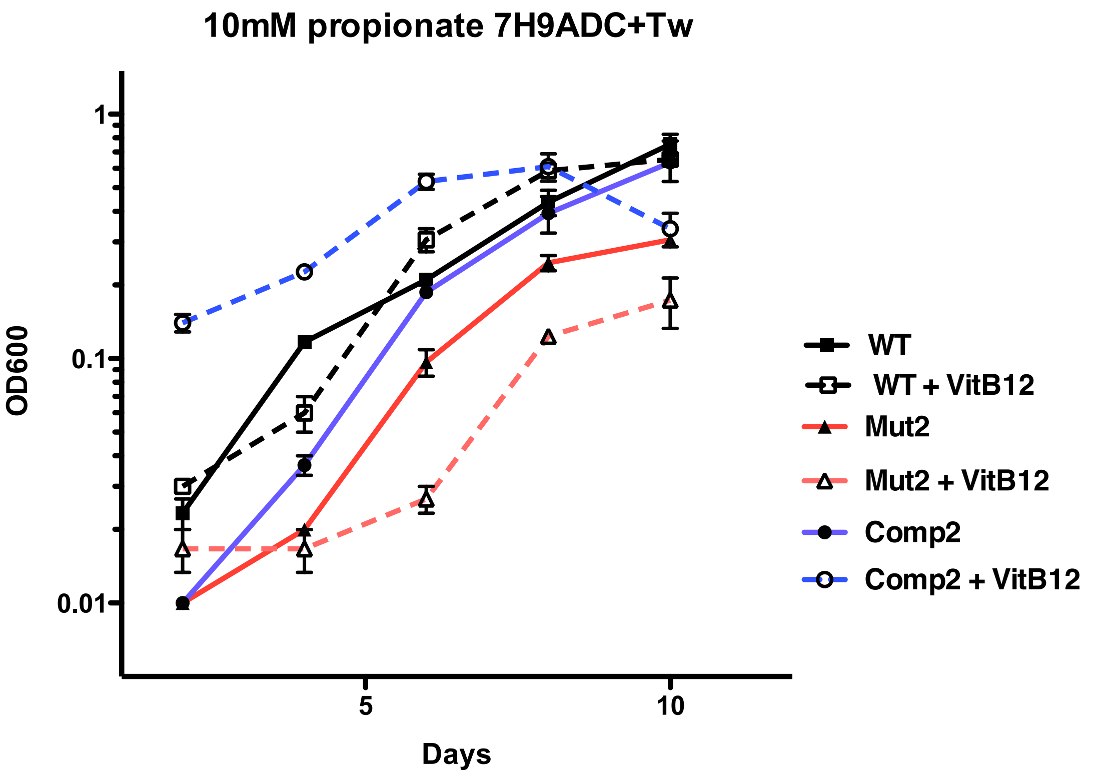

Figure S9 (related to Figure 6). Vitamin B12 supplementation does not rescue growth defect of LprG-Rv1410 mutant in propionate. The indicated strains were cultured in 10 mM propionate as described in the materials and methods with or without the addition of +/- 0.5 µg/mL vitamin B12 (VitB12; dashed lines) at 37°C with shaking. Growth measured by OD 600, mean +/- standard deviation.
