# Supplementary material for: Mycobacterial Metabolic Syndrome: LprG and Rv1410 Regulate Triacylglyceride Levels, Growth Rate and Virulence in Mycobacterium tuberculosis
Source: PLoS Pathog. 2016 Jan 11;12(1):e1005351. doi: 10.1371/journal.ppat.1005351 (PMC4709180; doi:10.1371/journal.ppat.1005351)
Supplement: S10 Fig — MIC was determined as described in the Supplementary Materials and Methods. The MIC for WT, Mut2, and Comp2 was 62.5–125μg/mL in 7H9+ OADC (Sigma). (PDF) [file ppat.1005351.s011.pdf]

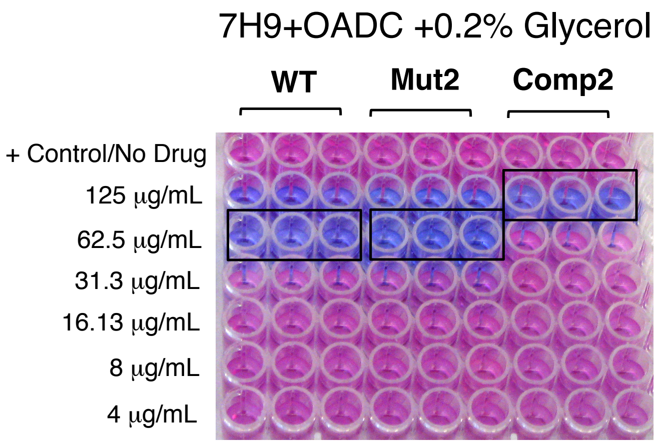

Figure S10 (related to Figure 6). Addition of glycerol decreases susceptibility of the LprG-Rv1410 mutant to tetrahydrolipostatin. MIC was performed as described in the Supplementary Materials and Methods. The MIC for WT, Mut2, and Comp2 was 62.5-125µg/mL in 7H9+ OADC (Sigma).
