# Supplementary material for: Mycobacterial Metabolic Syndrome: LprG and Rv1410 Regulate Triacylglyceride Levels, Growth Rate and Virulence in Mycobacterium tuberculosis
Source: PLoS Pathog. 2016 Jan 11;12(1):e1005351. doi: 10.1371/journal.ppat.1005351 (PMC4709180; doi:10.1371/journal.ppat.1005351)
Supplement: S1 Table — (PDF) [file ppat.1005351.s012.pdf]

Table S1 (related to Figure 4,5). Mouse strains used for competition and survival experiments.

Name	Defect	Strain	Source
Bl6	None (wild type)	C57Bl/6J	Jackson
nos2-/-	Inducible nitric oxide synthase	B6.129P2-*Nos2*^{tm1Lau}/J	Jackson
phox-/-	NADPH oxidase	B6.129S6-*Cybb*^{tm1Din}/J	Jackson
inf-γ-/-	IFNγ	B6.129S7-*Infγ*^{tm1Ts}/J	Jackson
rag1-/-	VDJ recombination; no mature B and T lymphocytes	B6.129S7-*Rag1*^{tm1Mom}/J	Jackson
SCID	Severe combined immunodeficiency; no functional B and T lymphocytes	B6.CB17-*Prkdc*^{scid}/SzJ	Jackson
