# Supplementary material for: Mycobacterial Metabolic Syndrome: LprG and Rv1410 Regulate Triacylglyceride Levels, Growth Rate and Virulence in Mycobacterium tuberculosis
Source: PLoS Pathog. 2016 Jan 11;12(1):e1005351. doi: 10.1371/journal.ppat.1005351 (PMC4709180; doi:10.1371/journal.ppat.1005351)
Supplement: S2 Table — (PDF) [file ppat.1005351.s013.pdf]

Table S2 (related to Figure 6): Effect of Carbon source restriction of growth rate of *Mtb in vitro*.

Carbon Source	Average Generation Time (hours)			
	WT (+/- SD)	Mut2 (+/- SD)	Comp2 (+/- SD)	p-value Mut2 v WT; Mut2 v Comp2
0.01% Cholesterol (Sauton's)	20.63 +/- 1.27	56.93 +/- 7.44	24.18 +/- 2.58	**0.006; 0.002**
0.2% Glycerol (7H9)	17.08 +/- 0.99	13.40 +/- 0.06	14.80 +/- 0.84	0.373; 0.067
10mM Propionate (Sauton's)	170.95 +/- 3.77	390.73 +/- 85.69	124.48 +/- 4.77	**0.008; 0.003**
10mM Propionate (7H9)	89.86 +/- 6.09	148.29 +/- 25.94	66.42 +/- 4.06	**0.004; 0.004**
0.2% Glycerol, 10mM Propionate (7H9)	30.48 +/- 1.00	27.69 +/- 0.12	31.02 +/- 1.04	0.062; **0.035**
10mM Propionate, 5 mM Acetate (Sauton's)	43.96 +/- 1.56	51.74 +/- 3.51	39.57 +/- 1.27	**0.025, 0.003**
10mM Propionate, 5mM Acetate (7H9)	40.85 +/- 0.93	35.72 +/- 1.09	26.19 +/- 0.91	**0.007; 0.009**
0.2% Glycerol, 10mM Propionate, 5mM Acetate (7H9)	28.41 +/- 0.61	27.45 +/- 0.97	29.13 +/- 0.59	0.336; 0.183
Oleic acid, 0.2% Glycerol, 10mM Propionate, 5mM Acetate	25.11 +/- 0.4	25.16 +/- 0.77	25.78 +/- 0.65	0.884; 0.528

Growth rate was defined as doubling time as estimated from the average slope of the log-linear portion of the growth curve as follows: an exponential line was fitted to the average LOG2(OD600) values of the log-linear curves in triplicate. The average slope was derived from the triplicate lines by the equation, $y=mx+b$ of the fitted line, with slope=m, and doubling time in hours as $1/m*24$. Student's two-tailed t-tests for slope of Mut2 versus WT and Mut2 versus Comp2 are reported.
